# New insights into the phylogeny of *Carasobarbus* Karaman, 1971 (Actinopterygii, Cyprinidae) with the description of three new species

**DOI:** 10.1038/s41598-024-71463-7

**Published:** 2024-09-18

**Authors:** Arash Jouladeh-Roudbar, Cüneyt Kaya, Saber Vatandoust, Hamid Reza Ghanavi

**Affiliations:** 1 Museum für Naturkunde, Leibniz Institute for Evolution and Biodiversity Science, 10115 Berlin, Germany; 2 Faculty of Fisheries, Recep Tayyip Erdogan University, Rize, Turkey; 3 Department of Fisheries, Babol Branch, Islamic Azad University, Babol, Iran; 4 Department of Biology, Lund University, Lund, Sweden

**Keywords:** Himri, Freshwater fish, Morphology, Integrative taxonomy, Western Asia, Phylogeny, Ichthyology, Biodiversity, Phylogenetics, Taxonomy

## Abstract

Fishes from the genus *Carasobarbus*, widely distributed throughout the river systems of North Africa and West Asia, are commonly referred to as Himris. In the Persian Gulf basin, they are widespread and are also found in fast-flowing rivers or the deeper regions of lakes. In this region, representation of these fishes in scientific collections is scarce, and except for *C. luteus*, the other species are very poorly documented and frequently misidentified due to their similarities. In this study we analysed the relationships among *Carasobarbus* species using mitochondrial genes (Cyt *b*, COI) and present morphological characters based on examinations. Our results revealed three new species which we describe here. *Carasobarbus doadrioi,* new species, is distinguished by 40–44 scales on the lateral line and a prominent black blotch on end of caudal peduncle in specimens < 85 mm SL. *Carasobarbus hajhosseini*, new species is distinguished by 32–34 scales on the lateral line and long head length (20–24% SL). *Carasobarbus saadatii*, new species, is distinguished by 38–40 scales on the lateral line and short head length (19–20% HL). In the Persian Gulf basin, *Carasobarbus* species exhibit uncorrected genetic distances of 1.6 to 5.5% in the COI barcode region and 2.6% to 9.9% in the Cyt *b* gene. This study highlights the importance of investigating the unexplored diversity that exists within poorly sampled and understudied freshwater fish group. Such investigations are essential for developing a comprehensive understanding of the true extent of biodiversity, which is critical for informing effective conservation and protection strategies.

## Introduction

*Carasobarbus* Karaman, 1971 is a small genus of Cyprinidae comprising 10 valid species distributed across Southwest Asia and Northwest Africa^1–3^. These fishes known as Himris and characterized by large scales and special forms of the lips^4,5^. Three species of Himris are currently known from the Persian Gulf basin: *Carasobarbus luteus* (Heckel, 1843), *C. kosswigi* (Ladiges, 1960), and *C. sublimus* (Coad & Najafpour, 1997), with the latter considered to be endemic to Iran. Initially, *C. kosswigi* was described as *Cyclocheilichthys* Bleeker, 1859, *C. sublimus* as *Barbus* Daudin 1805, and *C. luteus* as *Systomus* McClelland 1838. Bianco and Bănărescu^6^ considered *luteus* as *Carasobarbus* validating the genus. Karaman^7^ erected *Kosswigobarbus* and placed *kosswigi* in it. However, Borkenhagen et al.^8^ synonymized this genus with *Carasobarbus*. In the following, Borkenhagen and Krupp^2^ conducted a comprehensive taxonomic revision of the genus *Carasobarbus*, revealing three valid species inhabiting Iran: *C. luteus*, *C. kosswigi*, and *C. sublimus*. They mentioned that *C. sublimus* is present in Zohre and Karkheh drainages, and *C. kosswigi* is found in Karun and Tigris drainages.

The elusive nature of *Carasobarbus* species and the challenges associated with sampling them have rendered the study of these fishes extremely difficult. This is especially accentuated because some species are rare and easily misidentified with other species inhabiting the same habitats. After approximately 15 years of field expeditions across Iran, Iraq, and Türkiye, during which *Carasobarbus* specimens were collected from the type localities of *C. kosswigi* and *C. sublimus*, as well as other populations from the Tigris to Zohreh drainages, a comprehensive examination revealed significant morphological and genetic differences among them. Our findings provide evidence supporting the existence of three undescribed species in Iran, which we describe based on a combination of morphological and molecular genetic characters.

## Materials and methods

### Fish sampling and preservation

All fish specimens used in this study were sampled following local guidelines and rules. All experimental protocols are approved routine procedures by ethics committee in Lund University. All methods were carried out in accordance with relevant guidelines and regulations, and all methods are reported in accordance with ARRIVE guidelines. The sampling permits were issued by the local environment department. Fish were euthanized with an overdose of clove oil, fixed in 10% formalin for 24 h, and preserved in ethanol 70%. The samples used in molecular analyses were fixed in 99% EtOH (whole body or a fin clip).

### Morphological examination

**Fig. 1:**
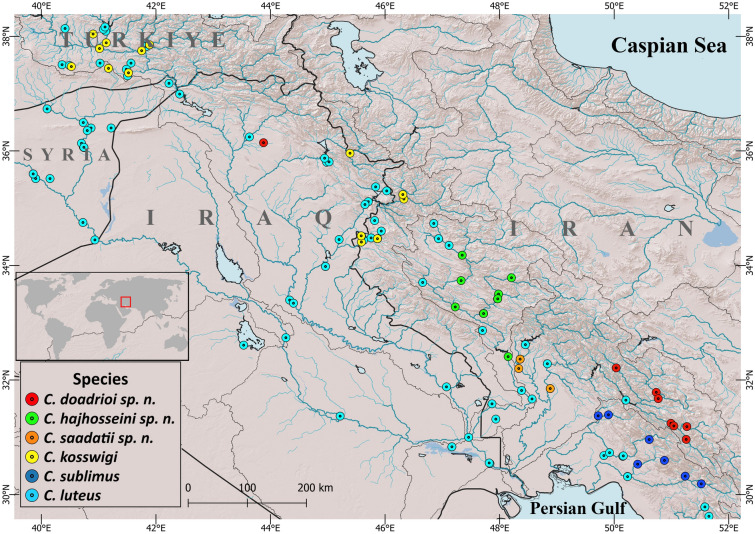
Distribution map of *Carasobarbus* species in Persian Gulf basin.

Measurements were made point-to-point with a digital calliper and recorded to 0.1 mm. Counts and measurements were made on the left side of specimens whenever possible, following Kottelat & Freyhof^9^. Head length and measurements of body parts are given as proportions of standard length (SL). Subunits of the head are presented as proportions of head length (HL). Standard length (SL) was measured from the tip of the snout to the posterior extremity of the hypural complex. The skin fold at the posterior part of the gill cover was included in the measurement of HL. The length of the caudal peduncle was measured from behind the base of the posterior anal-fin ray to the posterior extremity of the hypural complex, at mid-height of the caudal-fin base. The last two branched rays articulating on a single pterygiophore in the dorsal and anal-fins are noted as "11/2". The distribution map (Fig. 1) was created with QGIS v.3.18 software (http://qgis.org). In addition to examined specimens of *C. sublimus*, morphometric data were obtained from Coad and Najafpour^1^.

### DNA extraction, PCR amplification and sequencing

Genomic DNA was extracted using Macherey & Nagel NucleoSpin® Tissue kits following the provided protocol. The barcode region of the COI (cytochrome c oxidase subunit 1) gene was amplified using FishF1-5′TCAACCAACCACAAAGACATTGGCAC3′ and FishR1-5′TAGACTTCTGGGTGGCCAAAGAATCA3′^10^, and the Cyt *b* genetic marker using GluF-5’AACCACCGTTGTATTCAACTACAA3’ and ThrR5’ ACCTCCGATCTTCGGATTACAAGACCG3’^11^. The T7Promoter (5’TAATACGACTCACTATAGGG3’) and T3 (5’ATTAACCCTCACTAAAGGG3’) standard sequences were added to the sequence of forward and reverse primers respectively, to simplify the sequencing of different PCR products on the same plate. Sequencing of the PCR products was performed at an external sequencing service provider.

### Molecular data analysis

The obtained sequences and the ones downloaded from GenBank (Tables 1, 2), were aligned using MAFFT^12,13^ as implemented in Geneious v. 10.0.2 (Biomatters, http://www.geneious.com/). The obtained datasets were concatenated in Geneious to create three different datasets: COI dataset, Cyt *b* dataset, and the concatenated dataset. In the case of the concatenated dataset, in the ingroup, we only kept the samples with both genetic markers amplified from the same specimen. This was not possible for the outgroups as none of the sequences in Genbank, used for outgroups, came from the same specimen for both genes. In these cases, sequences from unrelated specimens were concatenated together. This does not affect the phylogenetic results of the ingroup. To determine intraspecific species uncorrected pairwise genetic distances (p-distances) (Tables 3, 4), we employed Mega 6^14^.

**Table 1:** GenBank accession numbers of the COI sequences downloaded for this study.

GenBank	Species	GenBank	Species
KJ552897	*C. canis*	KJ552960	*C. fritschii*
KJ552760	*C. canis*	KJ553111	*C. fritschii*
KJ552827	*C. canis*	KJ553144	*C. fritschii*
KJ553264	*C. chantrei*	KJ553161	*C. fritschii*
KJ553201	*C. chantrei*	KJ553212	*C. fritschii*
KJ553098	*C. chantrei*	KJ553240	*C. fritschii*
KJ553228	*C. chantrei*	KJ552291	*C. fritschii*
KJ552821	*C. chantrei*	KM590427	*C. hajhosseini*
KJ552958	*C. chantrei*	KM590426	*C. luteus*
KM590423	*C. doadrioi*	KM590424	*C. luteus*
MW250390	*C. doadrioi*	KM590425	*C. luteus*
KM590428	*C. kosswigi*	MW250388	*C. luteus*
KJ552798	*C. harterti*	OR038182	*C. luteus*
KJ552803	*C. harterti*	OR038183	*C. luteus*
KJ552814	*C. harterti*	OR038192	*C. luteus*
KJ552851	*C. harterti*	OR038193	*C. luteus*
KJ552906	*C. harterti*	OP456596	*Mesopotamichthys sharpeyi*
KJ552966	*C. harterti*	OP456597	*M. sharpeyi*
KJ552780	*C. fritschii*	OP456598	*M. sharpeyi*
KJ552819	*C. fritschii*	KM590450	*Arabibarbus grypus*
KJ552951	*C. fritschii*	KM590451	*A. grypus*
KJ552959	*C. fritschii*

**Table 2:** GenBank accession numbers of the Cyt *b* sequences downloaded for this study.

GenBank	Species	GenBank	Species
KU525007	*C apoensis*	KU524970	*C. fritschii*
KU525008	*C apoensis*	KU524973	*C. fritschii*
KU525009	*C apoensis*	KU524974	*C. fritschii*
KU525006	*C apoensis*	KU524969	*C. fritschii*
AF145947	*C. canis*	KU524971	*C. fritschii*
KU524924	*C. canis*	MN961175	*C. fritschii*
KU524925	*C. canis*	KU525005	*C. fritschii*
KU524926	*C. canis*	AF287430	*C. fritschii*
AF180852	*C. chantrei*	KU524978	*C. fritschii*
HQ167605	*C. chantrei*	KU524979	*C. fritschii*
KU524913	*C. chantrei*	KU524980	*C. fritschii*
KU524921	*C. chantrei*	KU524981	*C. fritschii*
KU524922	*C. chantrei*	KU524982	*C. fritschii*
KU524923	*C. chantrei*	KU524983	*C. fritschii*
KU524958	*C. chantrei*	KU524984	*C. fritschii*
KU524959	*C. chantrei*	KU524985	*C. fritschii*
KU524914	*C. chantrei*	KU524986	*C. fritschii*
KU524934	*C. doadrioi*	KU524935	*C. hajhosseini*
KU524901	*C. exulatus*	AF180855	*C. harterti*
KU524902	*C. exulatus*	KU524975	*C. harterti*
KU524904	*C. exulatus*	KU524976	*C. harterti*
KU524907	*C. exulatus*	KU524977	*C. harterti*
KU524908	*C. exulatus*	KP712261	*C. kosswigi*
KU524903	*C. exulatus*	AF180853	*C. kosswigi*
KU524905	*C. exulatus*	KU524915	*C. luteus*
AF180856	*C. fritschii*	KU524964	*C. luteus*
MN961176	*C. fritschii*	KU524965	*C. luteus*
KU524990	*C. fritschii*	KU524912	*C. luteus*
KU524993	*C. fritschii*	KU524920	*C. luteus*
KU524987	*C. fritschii*	KU524928	*C. luteus*
KU524988	*C. fritschii*	KU524963	*C. luteus*
KU524989	*C. fritschii*	KP712262	*C. luteus*
KU524991	*C. fritschii*	KU524933	*C. luteus*
KU524992	*C. fritschii*	KU524927	*C. luteus*
KU524995	*C. fritschii*	KU524929	*C. luteus*
KU524999	*C. fritschii*	KU524909	*C. sublimus*
MN961177	*C. fritschii*	KU524931	*C. sublimus*
KU524994	*C. fritschii*	KU524930	*C. sublimus*
AF287429	*C. fritschii*	KU524910	*C. sublimus*
KU524968	*C. fritschii*	KU524911	*C. sublimus*
KU524996	*C. fritschii*	KU524932	*C. sublimus*
KU524997	*C. fritschii*	KF876032	*Mesopotamichthys sharpeyi*
KU525000	*C. fritschii*	KF876033	*M. sharpeyi*
KU525001	*C. fritschii*	KF876031	*M. sharpeyi*
KU525002	*C. fritschii*	KF876029	*Arabibarbus grypus*
KU525004	*C. fritschii*	KF876028	*A. grypus*
KU524966	*C. fritschii*	KF876021	*A. arabicus*
KU524967	*C. fritschii*	KF876022	*A. arabicus*
KU525003	*C. fritschii*	KF876023	*A. adharami*
KU524998	*C. fritschii*	KF876024	*A. adharami*
KU524972	*C. fritschii*

**Table 3:** Uncorrected-p genetic distances (%) in COI gene between different species of *Carasobarbus* (I.: intraspecific distance).

N	Species	I	1	2	3	4	5	6	7	8	9
1	*C. doadrioi* sp. n	0.06									
2	*C. hajhosseini* sp. n	0.00	4.1								
3	*C. saadatii* sp. n	0.09	2.5	4.7							
4	*C. canis*	0.00	5.0	5.3	4.7						
5	*C. chantrei*	0.21	4.9	5.3	5.0	2.0					
6	*C. kosswigi*	0.00	1.6	3.8	1.6	4.1	4.2				
7	*C. luteus*	0.19	5.3	5.3	5.4	2.8	1.6	4.7			
8	*C. sublimus*	0.16	3.9	3.3	5.1	5.3	5.5	4.2	5.4		
9	C. *harterti*/*fritschii* 1	0.72	4.2	4.2	4.2	4.7	4.0	3.8	4.9	5.3	
10	C. *harterti*/*fritschii* 2	0.55	5.2	5.1	4.8	4.4	4.1	4.5	4.8	5.4	2.6

**Table 4:** Uncorrected-p genetic distances (%) in Cyt *b* gene between different species of *Carasobarbus* (I.: intraspecific distance).

N	Species	I	1	2	3	4	5	6	7	8	9	10
1	*C. doadrioi* sp. n	0.32										
2	*C. hajhosseini* sp. n	0.20	8.5									
3	*C. saadatii* sp. n	0.32	4.6	7.6								
4	*C. canis*	0.04	7.6	8.2	6.5							
5	*C. chantrei*	0.10	7.1	8.0	6.4	3.2						
6	*C. exulatus*	0.10	7.2	7.9	6.5	3.0	2.6					
7	*C. fritschii*	0.66	8.3	9.9	8.8	6.6	7.1	6.9				
8	*C. harterti*	0.04	8.7	9.9	8.3	5.7	6.2	6.0	3.1			
9	*C. luteus*/*apoensis*	0.59	7.6	8.1	6.9	3.4	2.6	3.2	7.0	6.2		
10	*C. kosswigi*	0.56	4.2	7.1	3.8	6.3	5.6	5.6	8.3	8.5	6.0	
11	*C. sublimus*	0.34	9.0	5.0	8.8	9.2	9.2	8.6	9.4	9.7	8.8	7.9

Both maximum likelihood (ML) and Bayesian (BI) methods have been used to construct phylogenetic relationships of the group. In the case of ML approach, IQ-TREE 1.6.12^15,16^ were used. In this case, the optimal substitution model and the best partitioning scheme based on the codon information, was investigated using ModelFinder^17^ with the Bayesian information criterion (BIC). In the case of single marker datasets, the codon position information was provided, and in the concatenated dataset both codon position and gene separation were provided to the program. The bootstrap (− b 500) approximations was used to calculate support values^18^. FigTree 1.4.4 (http://tree.bio.ed.ac.uk/software/figtree/) was used to visualize the resulting trees. In the case of the BI approach, MrBayes 3.2.7^19^ were used with two parallel simultaneous analyses for 2 × 10^7^ generations, each with four MCMC chains, and sampling every 2000 generations. The initial 25% of generations were discarded as the burn-in. An rjMCMC^20^ approach was implemented using the nst = mixed command. The proper convergence of the runs was verified using Tracer 1.7^21^.

Three distance-based molecular species delimitation methods were used: automatic barcode gap discovery (ABGD)^22^, assemble species by automatic partitioning (ASAP)^23^, and Bayesian Poisson Tree Processes model (bPTP)^24^. The ABGD analysis were performed on its online webserver (https://bioinfo.mnhn.fr/abi/public/abgd/abgdweb.html), exploring a range of ABGD settings with a parameter range of Pmin = 0.001, Pmax = 0.1, and a gap width of 1.5 over ten steps. The ASAP analysis was also made, using Simple Distance (p-distances), via its web interface (https://bioinfo.mnhn.fr/abi/public/asap/asapweb.html). The bPTP analysis was run only on the in-group on the online implementation of it (https://species.h-its.org/) using default settings.

### ZooBank

This published work and the Nomenclatural Acts it contains have been registered in ZooBank with the LSID urn:lsid:zoobank.org:pub:891DC1CD-C71C-4783-B009-F3141E542A9D, urn:lsid:zoobank.org:act:9296233A-FA76-41E7-9814-7D22FCF37CDC, urn:lsid:zoobank.org:act:B056DC31-E811-4B1C-850B-0DE8767A07F5, and urn:lsid:zoobank.org:act:B4B0C801-0C84-4247-B488-767EC4307CF9.

## Results

We were able to generate 38 new sequences (22 COI + 16 Cyt *b*) for six species of *Carasobarbus* from Iran, Iraq and Türkiye, in addition to 173 sequences from NCBI GenBank (Tables 1 and 2). The final alignment for COI consisted of 770 base pairs, with 676 positions being constant, 88 being parsimony informative and 6 being singletons (calculated just between in-group species), and for Cyt *b* the alignment was 1143 base pairs, with 872 positions being constant, 240 being parsimony informative and 29 being singletons (calculated just between in-group species for both genes).

The COI gene of *Carasobarbus* displayed an interspecific uncorrected-*p* genetic distance of 1.6% between *C. luteus* and *C. chantrei* as well as *C. doadrioi* sp. n., *C. saadatii* sp. n. and *C. chantrei* to 5.5% between *C. sublimus*. Average intraspecific distance for *Carasobarbus* species was 0.20%, ranging from 0.0 in *C. canis*, *C. hajhosseini*, and *C. kosswigi* to 0.72% in clade 1 of *C. fritschii*/*harterti* species group (Table 3).

For the Cyt *b* gene, the genetic distances between species ranged from 2.6% between *C. luteus/apoensis*, *C. chantrei* and *C. exulatus* to 9.9% between *C. harterti* and *C. chantrei* as well as between *C. hajhosseini, C. fritschi* and *C. harterti*. Also, the average intraspecific distance was 0.30%, ranging from 0.04% in *C. canis* and *C. harterti* to 0.66% in *C. fritschii*. Table 4 shows the genetic distances between and within the *Carasobarbus* species for Cyt *b* gene.

**Fig. 2:**
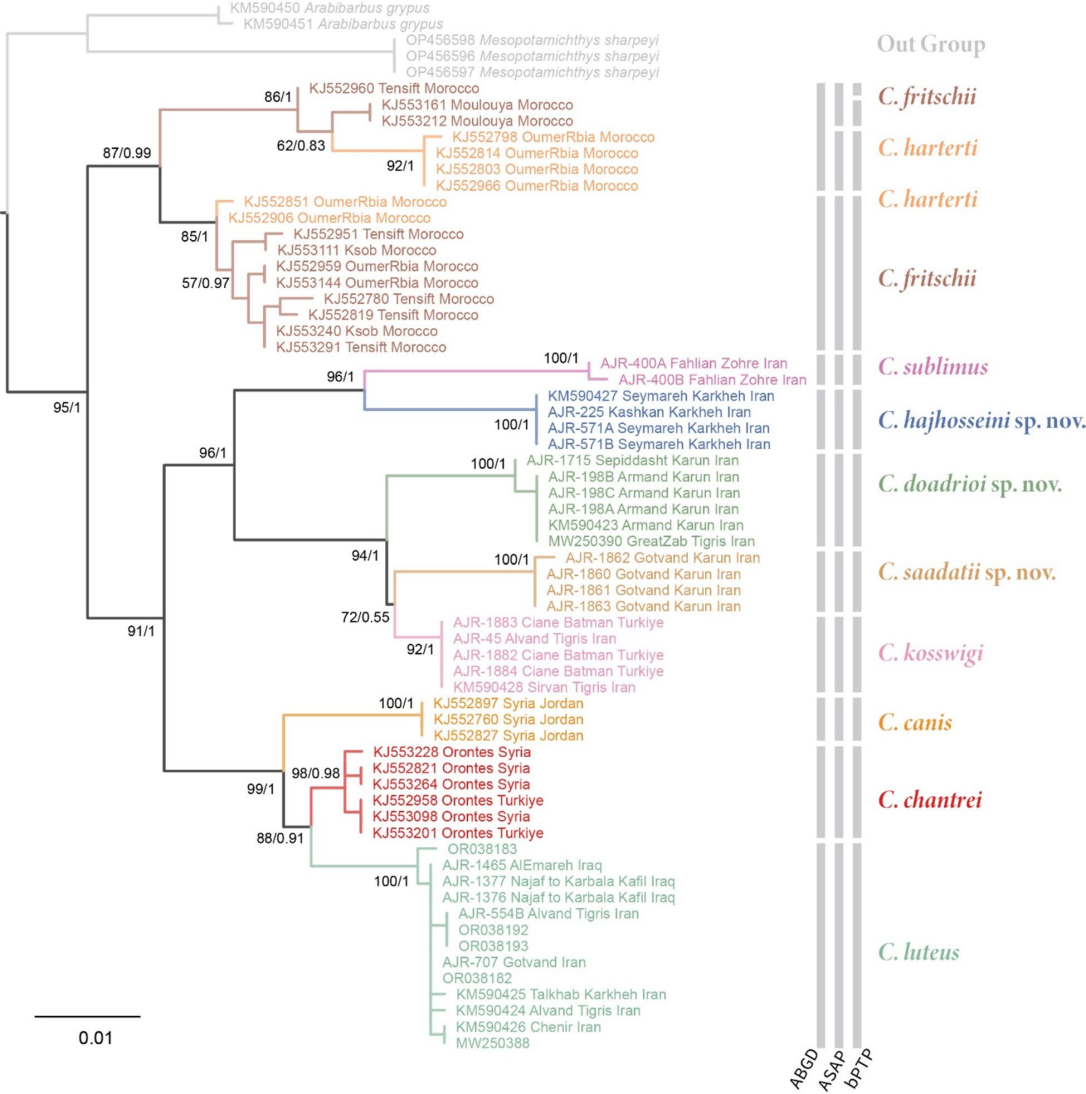
Phylogenetic tree of *Carasobarbus* based on the maximum likelihood and Bayesian analyses of the mitochondrial COI barcode region. Numbers present at each node are bootstrap/posterior probability support values. The result of the three different species delimitation methods is shown using the vertical bars.

**Fig. 3:**
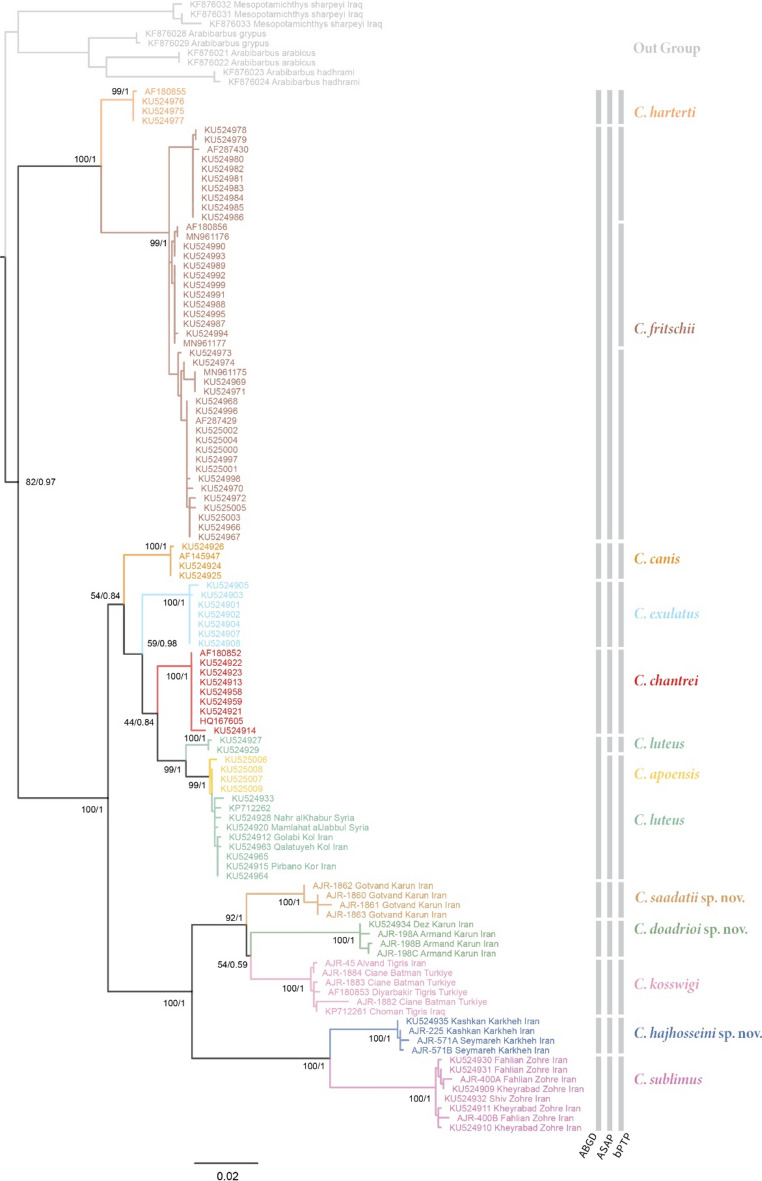
Phylogenetic tree of *Carasobarbus* based on the maximum likelihood and Bayesian analyses of the Cyt *b* gene. Numbers present at each node are bootstrap/posterior probability support values. The result of the three different species delimitation methods is shown using the vertical bars.

**Fig. 4:**
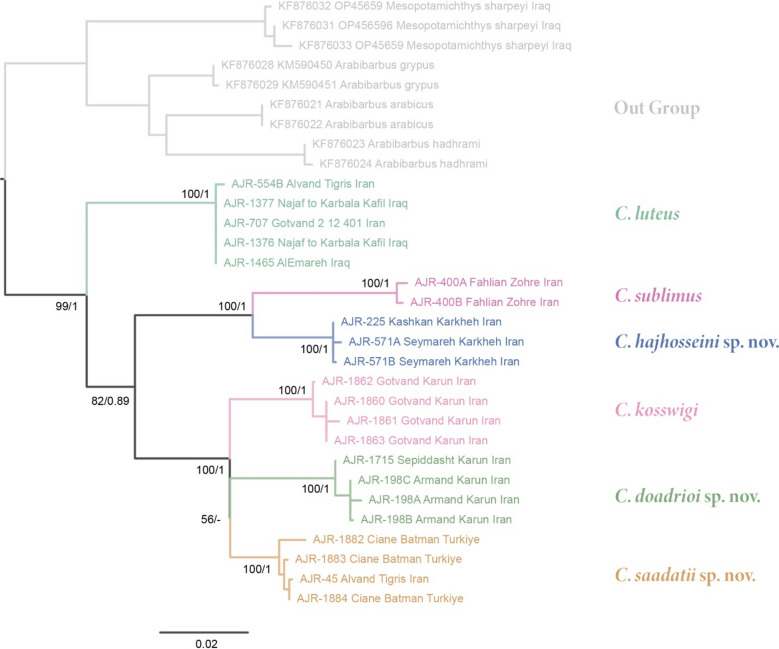
Phylogenetic tree of *Carasobarbus* based on the maximum likelihood and Bayesian analyses of the mitochondrial COI barcode region and the Cyt *b* markers concatenated. Numbers present at each node are bootstrap/posterior probability support values.

The general topology of Cyt *b*, COI and concatenated dataset trees (Figs. 2, 3 and 4) were in agreement with previously published phylogenies that focused on the genus *Carasobarbus*^8,25^. The COI and Cyt *b* dataset both resulted in acceptable trees with some nodes which were harder to resolve (not well supported). The increased sampling size, in the case of individual gene datasets, appears to improve the result compared to the prior phylogenetic works. The concatenation of the two genetic markers resulted in the best resolved tree even though the number of represented species was reduced. In general, all species analysed in any of the datasets was recovered as monophyletic apart from *C. harterti* and *C. fritschii* in the COI dataset. In this case, the resolution of the COI dataset for this part seems to not be adequate, and some samples identified as *C. harterti* are placed with *C. fritschii* and vice versa.

### Key to species of *Carasobarbus* in Persian Gulf basin

1a - Lower lip without median lobe; one pair of barbels (two pair in the Makran population).

………………*C. luteus*

1b - Lower lip with median lobe; two pair barbels.

………………2

2a - 24 − 29 total lateral-line scales; lower lip lobe well-developed (Coad and Najafpour, (1) data included).

………………*C*. *sublimus*

2b – 32–44 total lateral-line scales; lower lip lobe slightly to relatively developed.

.………………3

3a - 32–37 total lateral-line scales.

………………4

3b - 38–44 total lateral-line scales.

………………5

4a – Lower lip lobe well-developed; 32–77 [mode 36] total lateral-line scales; head length 25–27% SL; posterior barbel 13–20% HL; snout length 36–44% HL.

………………*C. kosswigi*

4b - Lower lip lobe slightly developed; 32–34 [mode 33–34] total lateral-line scales; head length 20–24% SL; posterior barbel 21–38% HL; snout length 25–31% HL.

………………*C. hajhosseini* sp. n.

5a – A prominent black blotch on end of caudal peduncle in specimens < 85 mm SL; head length 22–25% SL; dorsal fin height 19–26% SL; distance between base of pelvic and anal fins 24–25% SL.

………………*C. doadrioi* sp. n.

5b – No black blotch on end of caudal peduncle in specimens < 85 mm SL; Head length 19–20% SL; dorsal fin height 26–30% SL; distance between base of pelvic and anal fins 26–28% SL.

………………*C. saadatii* sp. n.

**Fig. 5:**
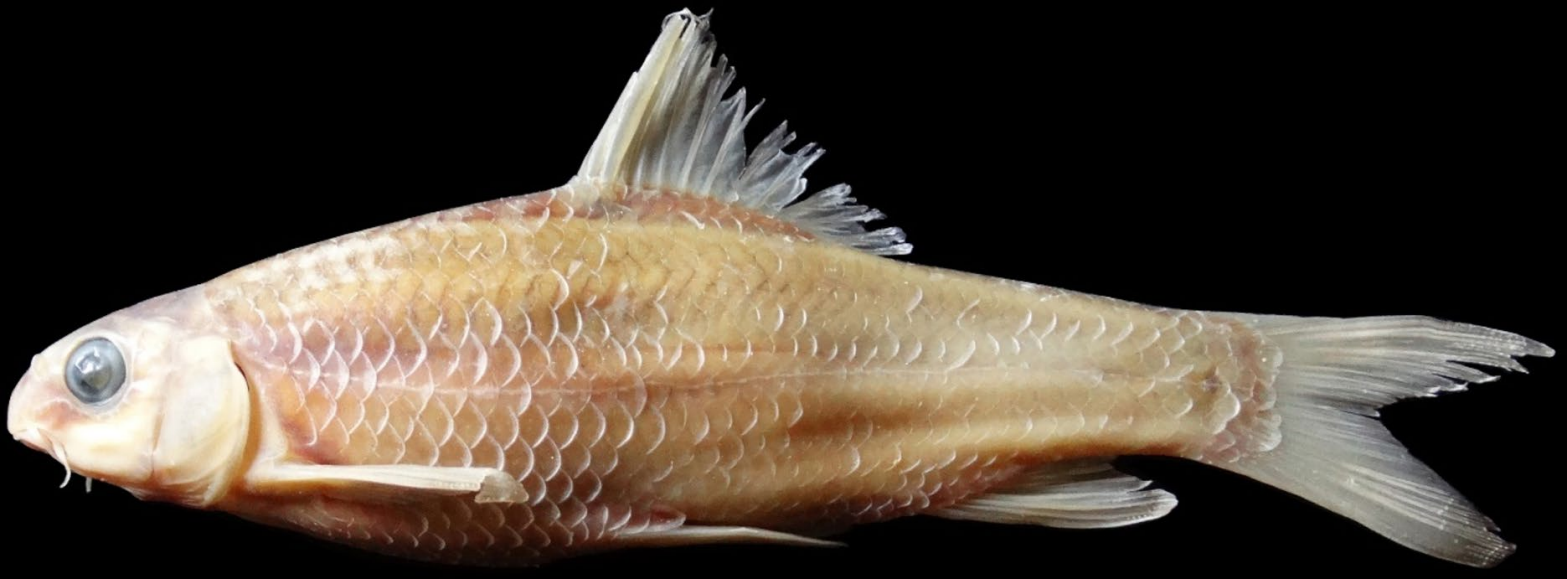
*Carasobarbus doadrioi* sp. n.; BIAUBM 6-H, holotype, 75 mm SL; Iran: Khersan River, Karun drainage.

**Fig. 6:**
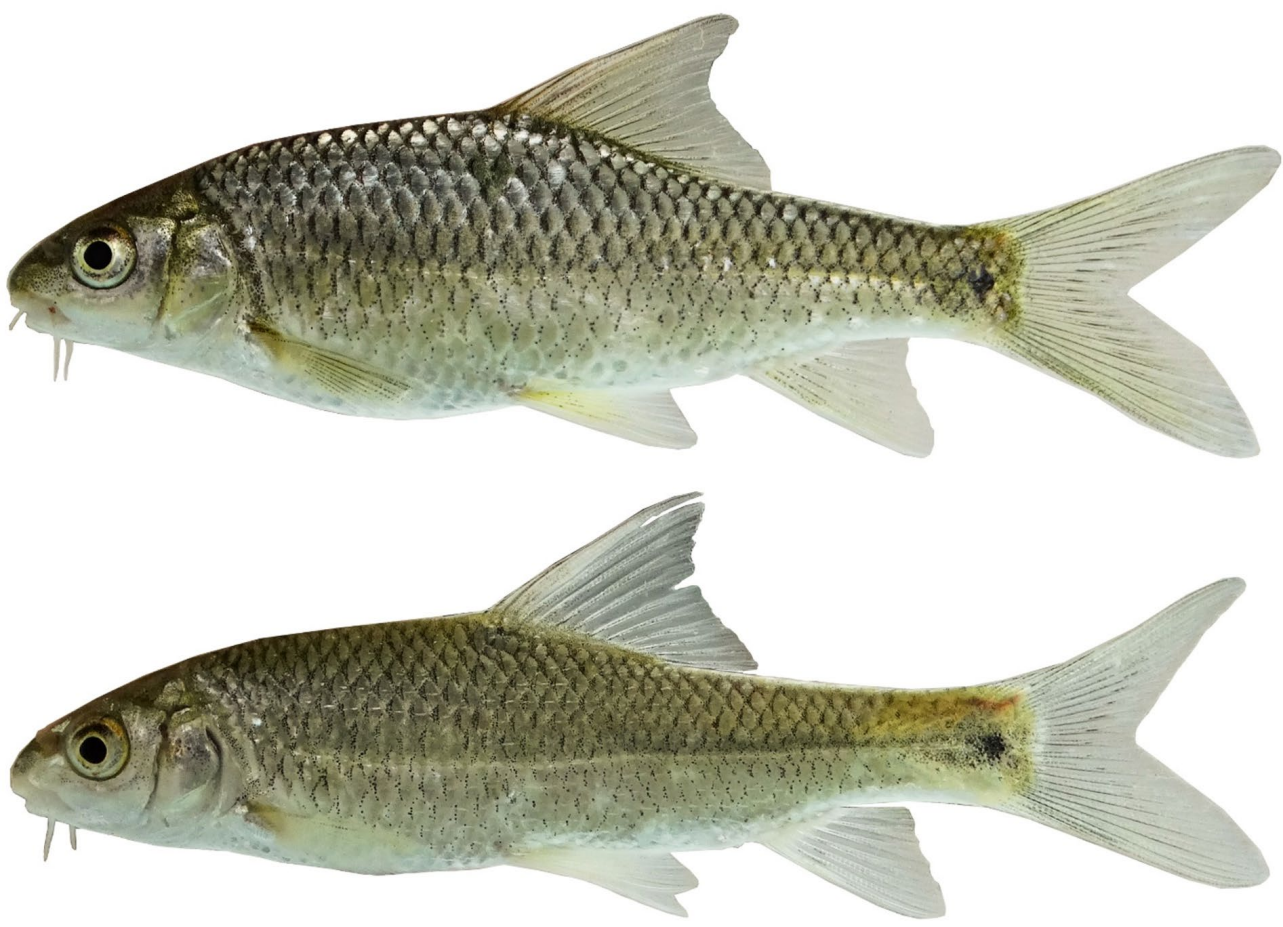
*Carasobarbus doadrioi* sp. n., AJRPC 17-P, paratypes, from top: 69 mm SL, 63 mm SL; Khersan River, Persian Gulf basin.

**Fig. 7:**
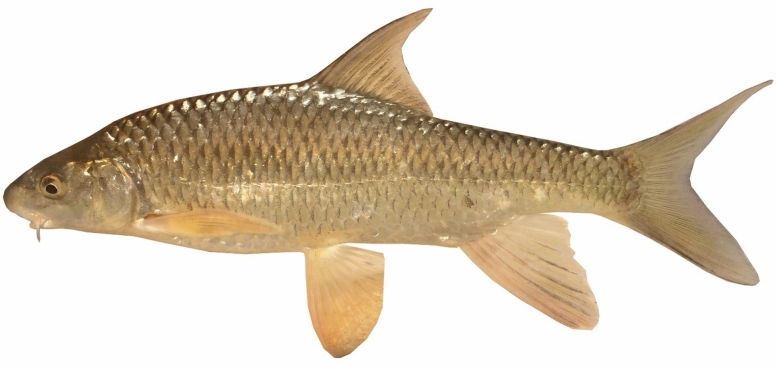
*Carasobarbus doadrioi* sp. n.; uncatalogued, about 150 mm SL; Iran: Khersan River, Karun drainage.

**Fig. 8:**
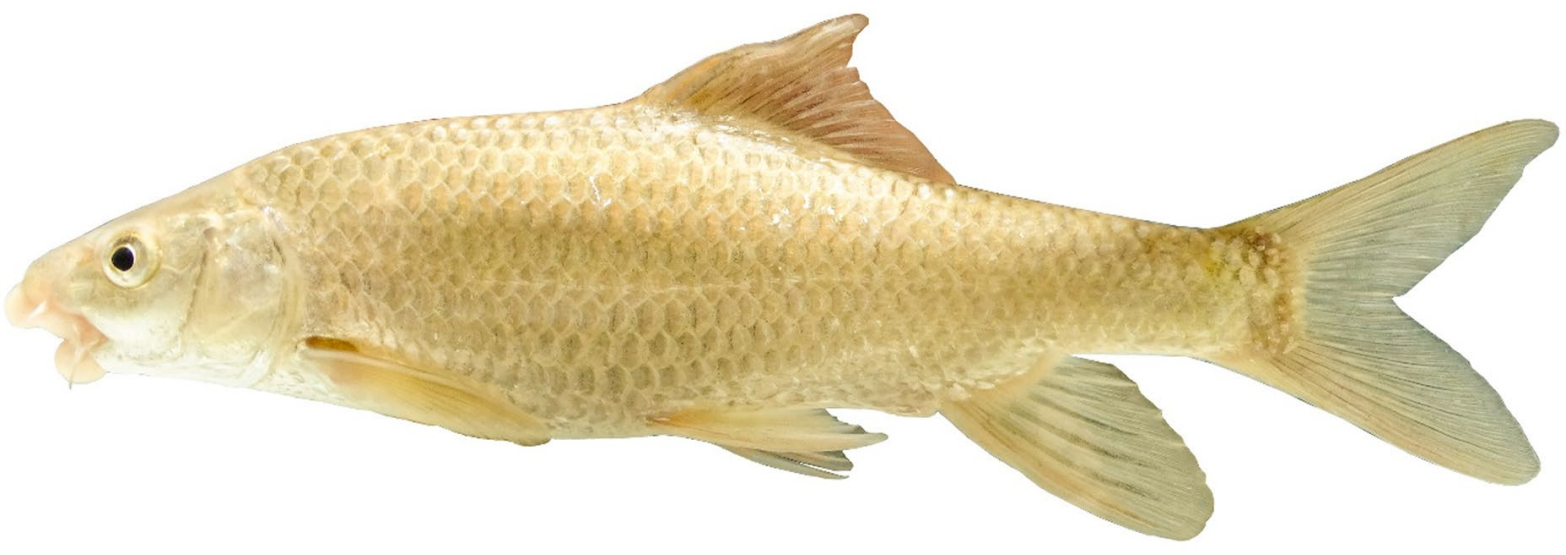
*Carasobarbus doadrioi* sp. n.; uncatalogued, about 150 mm SL; Iran: Khersan River, Karun drainage.

***Carasobarbus doadrioi*** **, new species**

(Figs. 5, 6, 7 and 8).

**Holotype**. BIAUBM 6-H, 75.3 mm SL; Iran: Chaharmahal and Bakhtiari prov., Khersan River at Atishgah, Karun River drainage, Persian Gulf Basin, 31.24358, 50.99075.

**Paratypes.** AJRPC 17-P, 7, 69.3–45.2 mm SL; data same as holotype.

**New material used in molecular genetic analysis.** AJRPC-DNA 198A (COI: PP515175, Cyt *b*: PP548209), 198B (COI: PP515176, Cyt *b*: PP548210), 198C (COI: PP515177, Cyt *b*: PP548211), same data as holotype; AJRPC-DNA 1715 (COI: PP515188, Cyt *b*: not sequenced), Iran: Lorestan prov., Sezar River at Absardeh, Karun River drainage, Persian Gulf Basin, 33.20562, 48.88326.

### Diagnosis

*Carasobarbus doadrioi* is distinguished from *C. sublimus*, *C. hajhosseini* sp. n. and *C. kosswigi* by having more scales on lateral line (40–44 vs. 27–37). *Carasobarbus doadrioi* sp. n. is similar to *C. saadatii* sp. n. and is distinguished by having a prominent black blotch on end of caudal peduncle in specimens < 85 mm SL (vs. no black blotch), longer head length (22–25 vs. 19–20% SL), shorter dorsal fin height (19–26 vs. 26–30% SL) and shorter distance between base of pelvic and anal fins (24–25 v. 26–28% SL). It is distinguished from *C*. *luteus* by having two pair of barbels (vs. one pair), well-developed median lobe on the lower lip (vs. without median lobe) and more scales on the lateral line (40–44 vs. 25–30) (Table 5).

**Table 5:** Lateral line scale count.

Species	25	26	27	28	29	30	31	32	33	34	35	36	37	38	39	40	41	42	43	44
*C. luteus*	1	2	**3**		1	1														
*C. hajhosseini* sp. n								3	**4**	**4**										
*C. kosswigi*								1	2	3	3	**6**	1							
*C. saadatii* sp. n														1	**2**	**2**				
*C. doadrioi* sp. n																**3**	1	1		1
*C. sublimus*			1	1	**2**															

Significant values are bold.

### Description

See Figs. 5, 6, 7 and 8 for general appearance, Table 6 for morphometric data. Body moderately high, laterally compressed, without nuchal hump. The greatest body depth in front or at dorsal-fin origin. Ventral head profile straight, dorsal head profile with a slight to pronounced hump near nostrils. Head short and narrow. Maximum body depth larger than head length. Triangular axillary scale at pelvic-fin base present. Pelvic-fin origin below vertical of last unbranched or first branched dorsal-fin ray. Caudal fin forked. Pectoral fin reaching approximately 70–90% of distance between pectoral- to pelvic-fin origin. Pelvic fin not reaching anus. Eye large, markedly smaller than snout. Mouth inferior, lips thick and fleshy with a well-developed median lob. Two pairs of barbels, rostral barbel reaches to anterior part of eye and maxillary barbel reaching to posterior part of eye.

**Table 6:** Morphometric data of *C. hajhosseini* sp. n. (holotype BIAUBM 7-H and paratypes AJRPC 18-P to 23-P; n = 11) and *C. doadrioi* sp. n. (holotype BIAUBM 6-H and paratypes AJRPC 17-P; n = 8).

Characters	*C. hajhosseini*					*C. doadrioi*				
	Holotype and paratypes					Holotype and paratypes				
	H	Min	Max	Mean	SD	H	Min	Max	Mean	SD
Standard length (SL)	191	86	184			75	45	69		
In percent of standard length										
Head length	22.5	19.8	24.1	22.3	1.4	22.1	22.1	24.7	23.5	1.2
Body depth at dorsal fin origin	30.8	26.6	32.9	30.2	1.6	29.9	26.2	30.3	28.3	1.6
Body depth at anal fin origin	21.0	19.0	22.4	20.9	1.1	20.8	16.7	20.8	19.2	1.5
Pre-dorsal length	53.5	50.4	57.1	53.4	2.1	50.0	50.0	52.3	51.1	0.9
Pre-pelvic length	53.2	49.4	53.6	51.2	1.3	51.3	49.1	53.4	51.4	1.7
Pre-anal length	78.5	74.6	78.7	76.6	1.6	76.0	73.7	76.0	75.2	0.9
Dis. betw. pectoral and anal fins	56.3	50.8	58.9	54.2	2.3	55.8	49.2	55.8	52.0	2.2
Dis. betw. pectoral and pelvic fins	30.2	25.5	31.0	28.6	1.7	31.4	25.6	31.4	28.0	2.0
Dis. betw. pelvic and anal fins	27.2	23.9	28.4	25.9	1.3	24.2	24.1	25.2	24.6	0.5
Dorsal fin height	23.3	22.1	28.2	24.8	1.9	18.8	18.8	25.6	23.1	2.3
Anal fin height	23.9	19.1	27.2	23.0	2.6	20.6	19.7	25.4	21.8	2.2
Pectoral fin length	20.7	19.9	25.6	22.7	1.9	24.0	20.8	24.0	22.1	1.3
Pelvic fin length	18.1	18.1	22.5	20.1	1.6	18.0	17.0	21.1	18.9	1.4
Upper caudal fin lobe	28.8	28.6	36.2	31.4	2.4	28.7	28.7	32.8	30.9	1.6
Length of middle caudal fin	12.0	11.3	14.4	12.9	1.0	13.8	12.0	16.1	14.0	1.6
Caudal peduncle length	15.7	14.7	18.0	15.9	1.2	15.5	14.8	16.4	15.4	0.6
Caudal peduncle depth	11.7	11.1	13.5	12.4	0.8	12.3	10.8	12.3	11.4	0.6
In percent of head length										
Snout length	31	25	31	27.4	2.2	26	22	29	25.5	2.2
Eye diameter	21	21	26	23.1	1.3	24	20	28	24.2	3.0
Head depth at pupil	78	56	79	65.5	8.7	59	54	59	56.2	2.1
Head depth at nape	88	81	96	87.0	4.2	85	72	85	78.9	4.8
Posterior barbel	25	21	38	27.7	5.4	17	17	24	21.3	2.2
Anterior barbel	13	13	19	16.9	2.3	20	13	20	15.2	2.9

Dorsal fin with 4 (n = 8) unbranched rays and 11½ (n = 8) branched rays, outer margin deeply concave. Anal fin with 3 (n = 8) unbranched and 6½ (n = 8) branched rays, outer margin straight. Pectoral fin with 14 (n = 5), 15 (n = 3) rays. Pelvic fin with 7 (n = 1)–8 (n = 7) rays. Lateral line with 40 (n = 3), 41 (n = 2), 42 (n = 1), 43 (n = 1), 44 (n = 1) scales. Scale rows between dorsal-fin origin and lateral line 7 (n = 8). Scale rows between anal-fin origin and lateral line 6 (n = 11).

### Coloration

In life: Body silverish or cream-white. Back darker than belly. Series of scales over the lateral line outlined by dark pigmentation, evident in anterior and fade in posterior. Fins with scattered dark melanophores on rays and membranes. In formalin: Cream-brown, back darker than belly. Series of scales over the lateral line with dark anterior pigmentation, fading posteriorly. Fins with scattered dark melanophores on rays and membranes.

### Distribution

Known from the lower Dez and Karun drainages.

### Etymology

This species name derives from the name of the Spanish ichthyologist Ignacio Doadrio Villarejo, in honour of his invaluable contribution to the study of the fishes of the world.

### Habitat

**Fig. 9:**
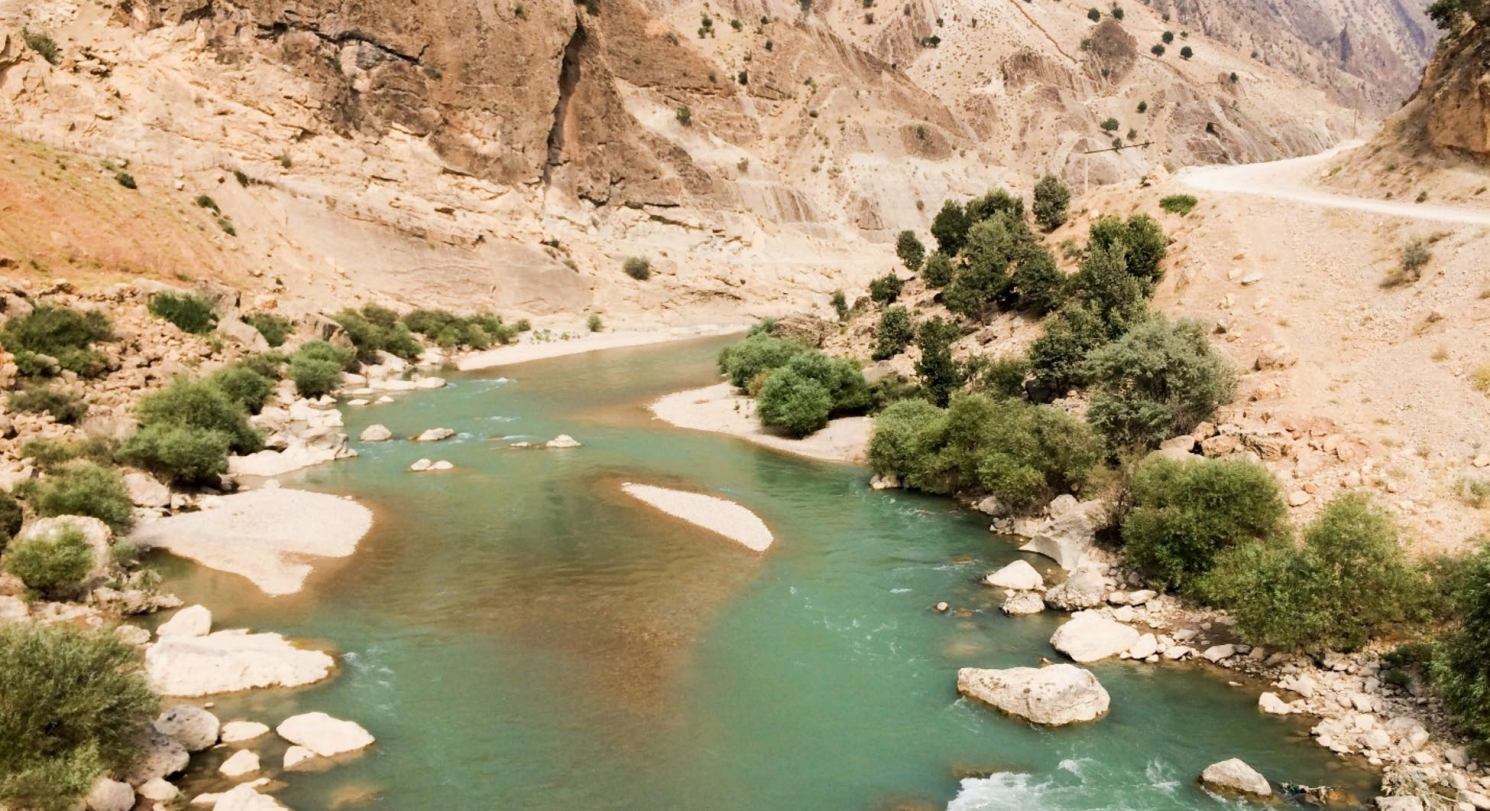
Khersan River at Atishgah, Karun drainage, type locality of *Carasobarbus doadrioi* sp. n.

*Carasobarbus doadrioi* sp. n. is found in the deep, slow current of large rivers (Fig. 9). It typically favours areas with abundant vegetation with rocky substrates during the summer. Generally, the species is most abundant in the middle and lower Karun drainage. *Luciobarbus esocinus* Heckel, 1843, *Garra rufa* (Heckel, 1843), *Acanthobrama marmid* Heckel, 1843, *Alburnus sellal* Heckel, 1843, *Chondrostoma regium* (Heckel, 1843), *Squalius berak* Heckel, 1843, *Oxynoemacheilus euphraticus*, *Glyptothorax cous* (Linnaeus 1766) and *G. alidaei* Mousavi-Sabet, Eagderi, Vatandoust & Freyhof, 2021 were found coexisting with the new species.

**Fig. 10:**
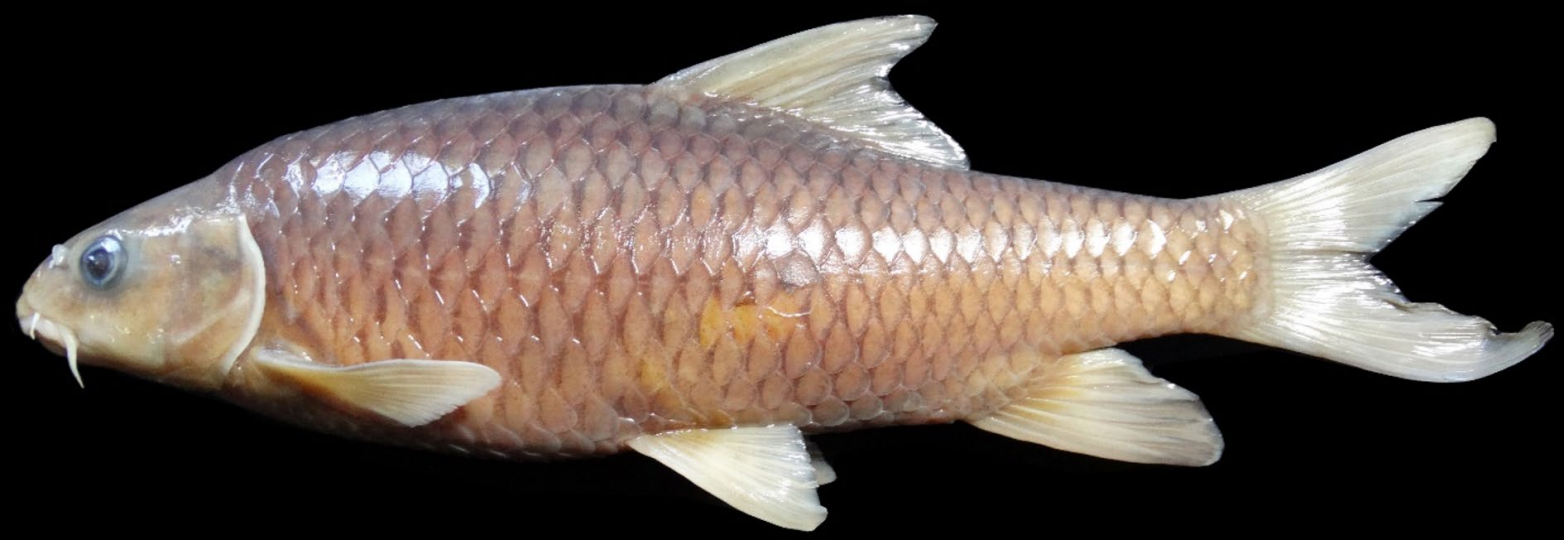
*Carasobarbus hajhosseini* sp. n.; BIAUBM 7-H, holotype, 191 mm SL; Iran: Seymareh River, Karkheh drainage.

**Fig. 11:**
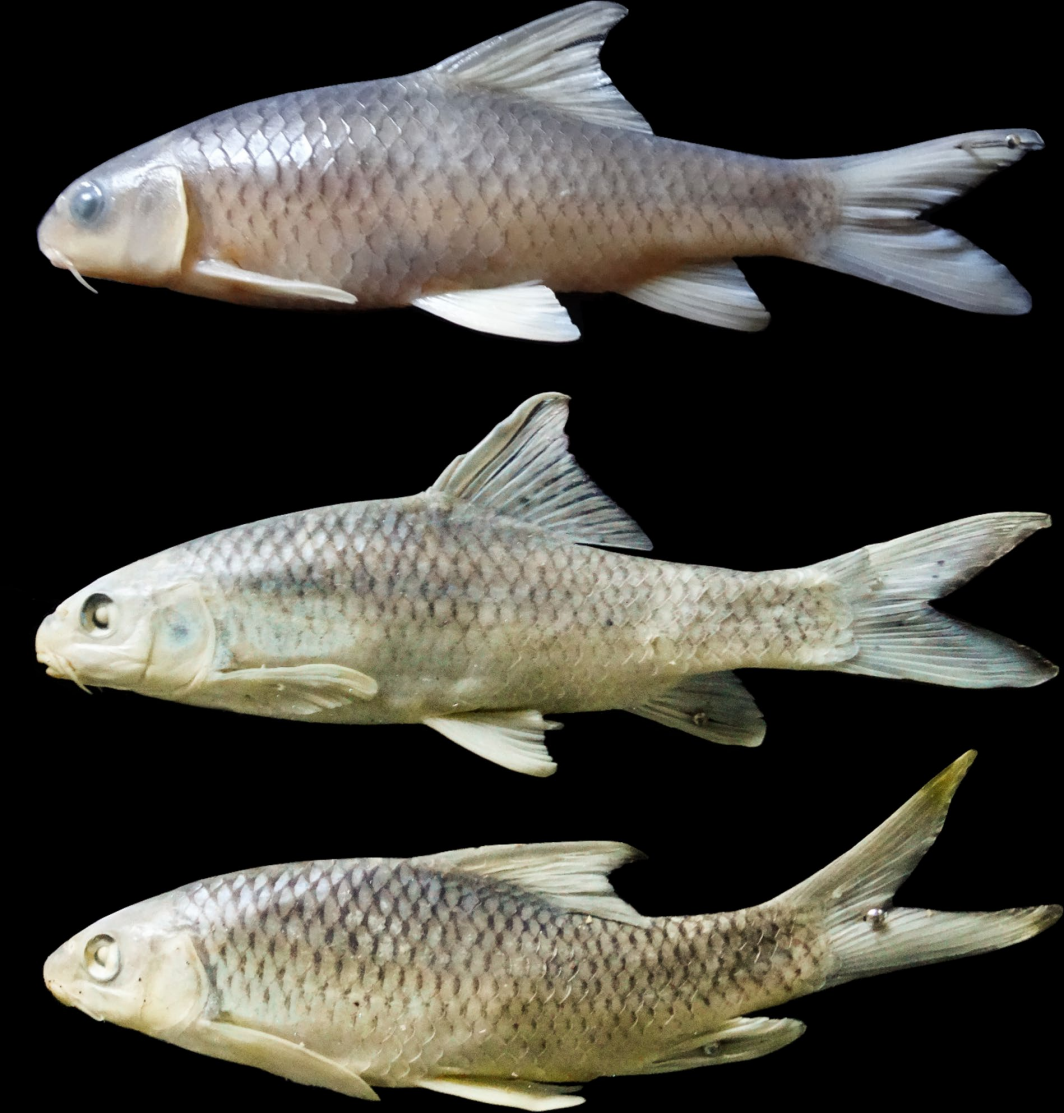
*Carasobarbus hajhosseini* sp. n., paratypes; from top: AJRPC 19-P, 109 mm SL; AJRPC 22-P, 113 mm SL; AJRPC 23-P, 94 mm SL; Iran: Karkheh drainage.

**Fig. 12:**
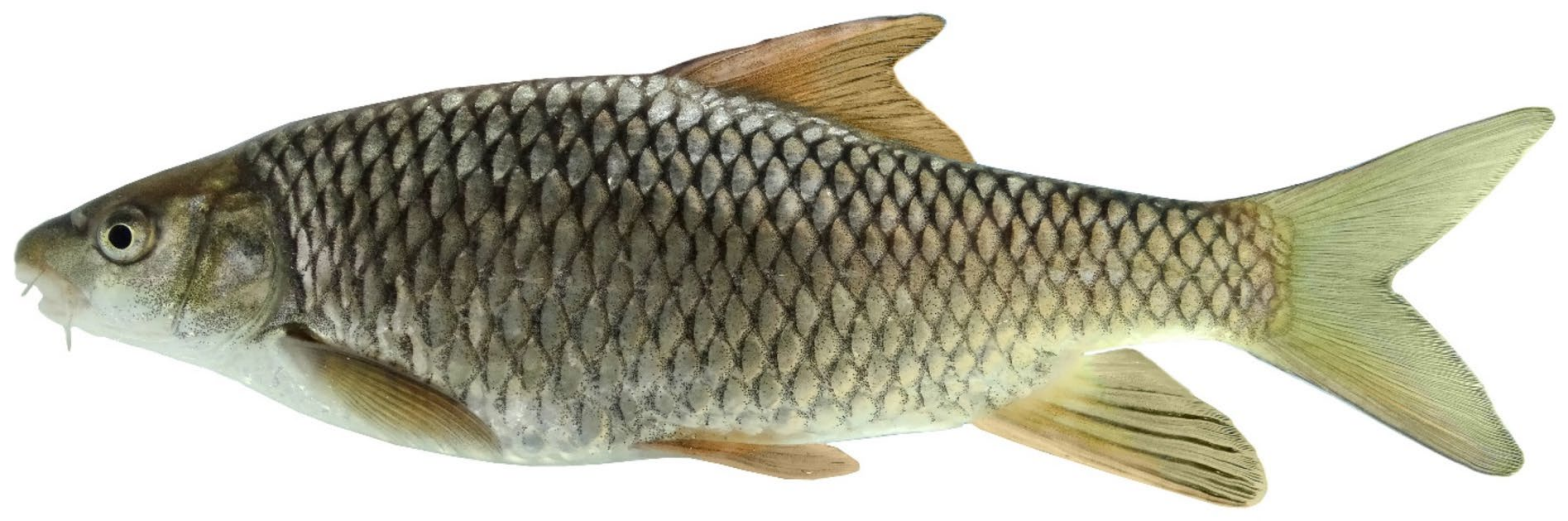
*Carasobarbus hajhosseini* sp. n.; BIAUBM 7-H, holotype, 191 mm SL; Iran: Seymareh River, Karkheh drainage.

**Fig. 13:**
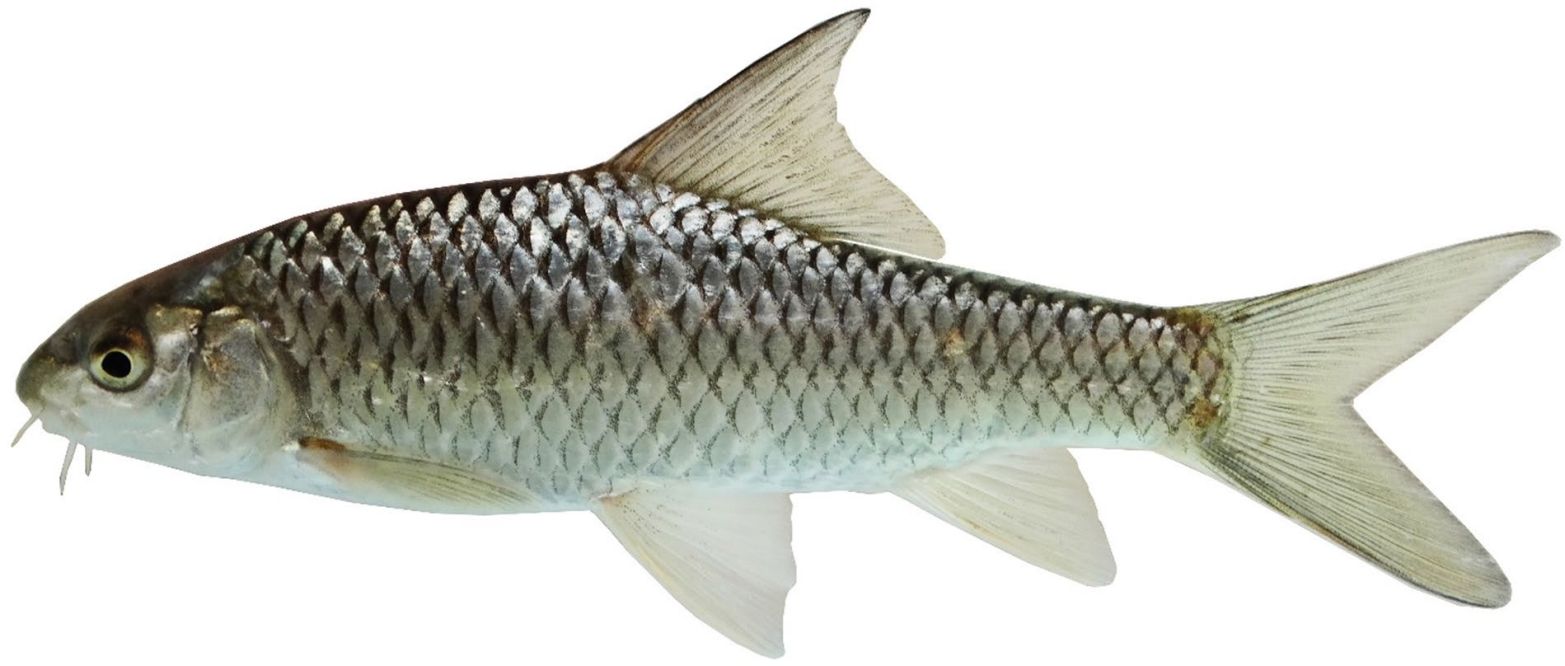
*Carasobarbus hajhosseini* sp. n.; AJRPC 19-P, paratype, 109 mm SL; Iran: Kahman River, Karkheh drainage.

***Carasobarbus hajhosseini*** **, new species**

(Figs. 10, 11, 12 and 13).

**Holotype**. BIAUBM 7-H, 190.6 mm SL; Iran: Ilam prov. Seymareh River at Talkhab, Karkheh drainage, Persian Gulf basin, 33.27771, 47.21252.

**Paratypes.** AJRPC 18-P, 4, 85.8–184.3 mm SL; same data as holotype. AJRPC 19-P, 2, 95.0–108.9 mm SL; Iran: Lorestan prov. Kahman River at Doab, Karkheh drainage, Persian Gulf basin, 33.78557, 48.20640. AJRPC 20-P, 1, 117.5 mm SL; Iran: Lorestan prov. Karkheh River at Pa Alam, Karkheh drainage, Persian Gulf basin, 32.83141, 48.03337. AJRPC 21-P, 1, 136.9 mm SL; Iran: Lorestan prov. Karkheh River at Mamulan, Karkheh drainage, Persian Gulf basin, 33.37823, 47.95654. AJRPC 22-P, 1, 113.2 mm SL; Iran: Lorestan prov. Karkheh River at Kal Sefid, Karkheh drainage, Persian Gulf basin, 33.08346, 47.53871. AJRPC 23-P, 1, 93.7 mm SL; Iran: Ilam prov. Karkheh River at Pol Zaal, Karkheh drainage, Persian Gulf basin, 32.98729, 47.76504.

**New material used in molecular genetic analysis.** AJRPC-DNA 225 (COI: PP515178, Cyt *b*: PP548212), Iran: Lorestan prov. Kahman River at Doab, Karkheh drainage, Persian Gulf basin, 33.78557, 48.20640; AJRPC-DNA 571A (COI: PP515182, Cyt *b*: PP548215), 571B (COI: PP515183, Cyt *b*: PP548216) same data as holotype.

### Diagnosis

*Carasobarbus hajhosseini* sp. n. is distinguished from *C. sublimus, C. saadatii* sp. n. and *C. doadrioi* sp. n. by having more scales on lateral line (32–34 vs. 24–29 in *C. sublimus*; 40–44 in *C. doadrioi* sp. n.; 38–40 in *C. saadatii* sp. n.).

*Carasobarbus hajhosseini* sp. n. is similar to *C. kosswigi* but can be distinguished by slightly developed lower lip lobe (vs. well-developed), shorter head (20–24 vs. 24–27% SL), shorter posterior barbel (13–20 vs. 21–38% HL) and shorter snout (25–31 vs. 36–44% HL).

Also, the new species can be distinguished from *C*. *luteus* by having two pair of barbels (vs. one pair), well-developed median lobe on the lower lip (vs. without median lobe) and more scales on the lateral line (32–34 vs. 25–30).

### Description

See Figs. 10, 11, 12 and 13 for general appearance, Table 6 for morphometric data. Body moderately high, laterally compressed, without nuchal hump. The greatest body depth at a level in front of or point of dorsal fin origin. Ventral head profile straight, dorsal profile has a slight to pronounced hump near nostrils. Head short and narrow. Maximum body depth larger than head length. Triangular axillary scale at pelvic-fin base. Pelvic-fin origin below vertical of last unbranched dorsal fin ray. Caudal fin forked. Tip of anal fin, when pressed to body, reaching to hypural complex. Pectoral fin reaching approximately 70–90% distance from pectoral-fin origin to pelvic-fin origin. Pelvic fin not reaching anus. Eye large, but smaller than snout. Mouth inferior, lips thick and fleshy with a small median lob. Two pairs of barbels, rostral not/or reaches to anterior part of eye and maxillary reaching to the posterior part of eye.

Dorsal fin with 4 unbranched rays and 10½ (n = 6)–11½ (n = 5) branched rays, outer margin deeply concave. Anal fin with 3 (n = 11) unbranched and 6½ (n = 11) branched rays, outer margin straight. Pectoral fin with 13 (n = 4), 14 (n = 6), 15 (n = 1) rays. Pelvic fin with 8 (n = 7)–9 (n = 4) rays. Lateral line with 32 (n = 3), 33 (n = 4), 34 (n = 4) scales. Scale rows between dorsal-fin origin and lateral line 6 (n = 11). Scale rows between anal-fin origin and lateral line 5 (n = 11).

### Coloration

In fresh: Body silverish or cream-white. The back darker than the belly. Upper lateral line scales outlined by dark pigmentation, evident in anterior and fade in posterior. Fins with scattered dark melanophores on rays and membranes. In formalin: Body cream-brown, back darker than belly. Upper lateral line scales outlined by dark pigmentation, prominent in anterior section, fades towards posterior.

### Distribution

The new species is known from the Gamasiab, Kahman, Kashkan and Seymareh in Karkheh drainage.

### Etymology

The species is named in honour of Haj Hossein Javadi Pour (HHJP), who is the father of the first author of this study (AJR).

### Habitat

**Fig. 14:**
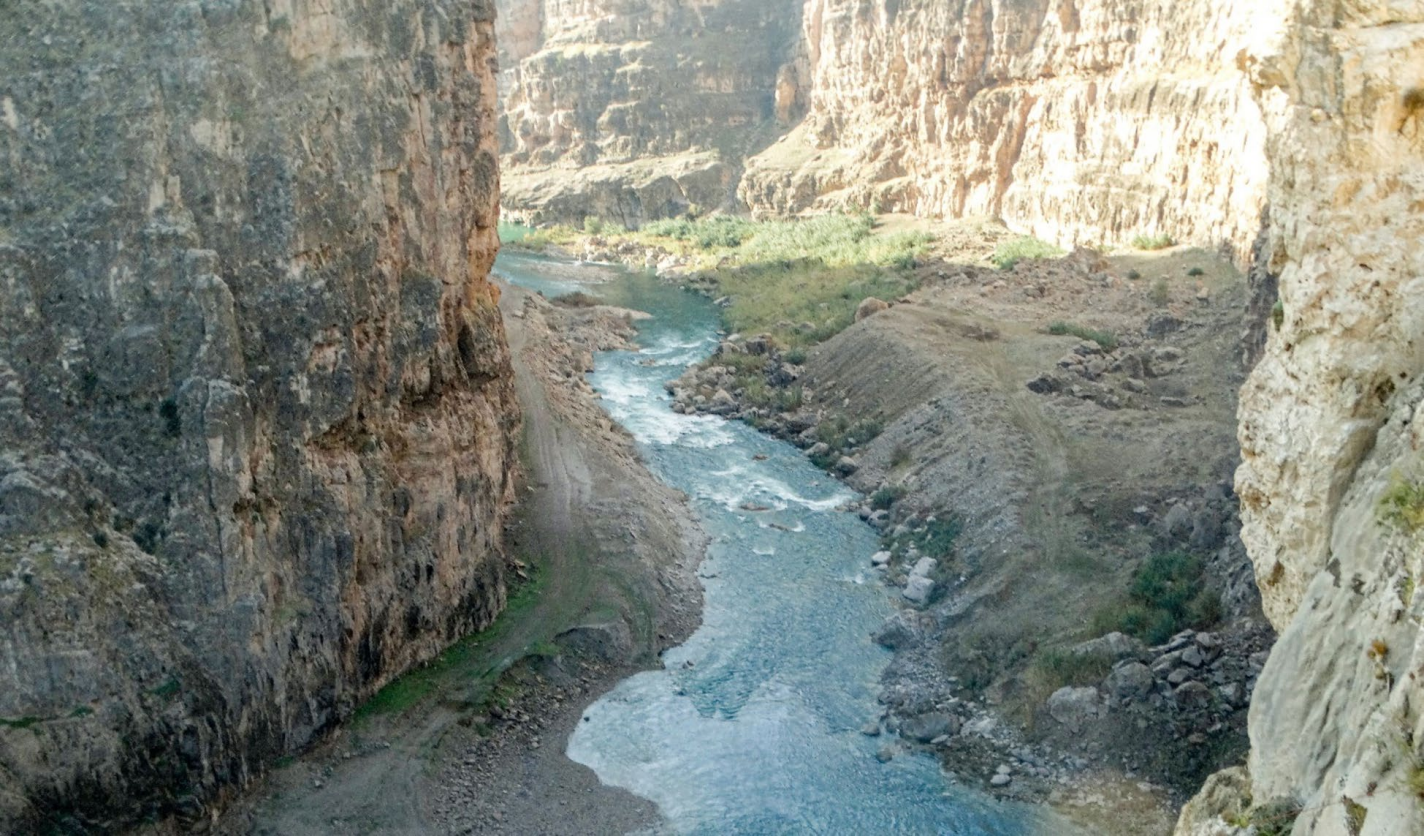
Seymareh River at Talkhab, Karkheh drainage, type locality of *Carasobarbus hajhosseini* sp. n.

*Carasobarbus hajhosseini* is commonly found in the deep, swiftly flowing sections of rivers and dam reservoirs (Fig. 14). It typically favours areas with abundant vegetation, and during the summer, it can also be observed in shallower waters. Generally, the species is most abundant in the middle and lower Karkheh drainage. *Luciobarbus esocinus*, *Capoeta* shajariani Jouladeh-Roudbar, Eagderi, Murillo-Ramos, Ghanavi & Doadrio, 2017, *Garra gymnothorax* Berg, 1949, *Chondrostoma regium*, *Alburnus sellal*, *Squalius lepidus* Heckel, 1843, *Squalius berak*, *Turcinoemacheilus saadii* Esmaeili, Sayyadzadeh, Özuluğ, Geiger & Freyhof, 2014, *Glyptothorax cous* and *G. alidaei* were found coexisting with the new species.

**Fig. 15:**
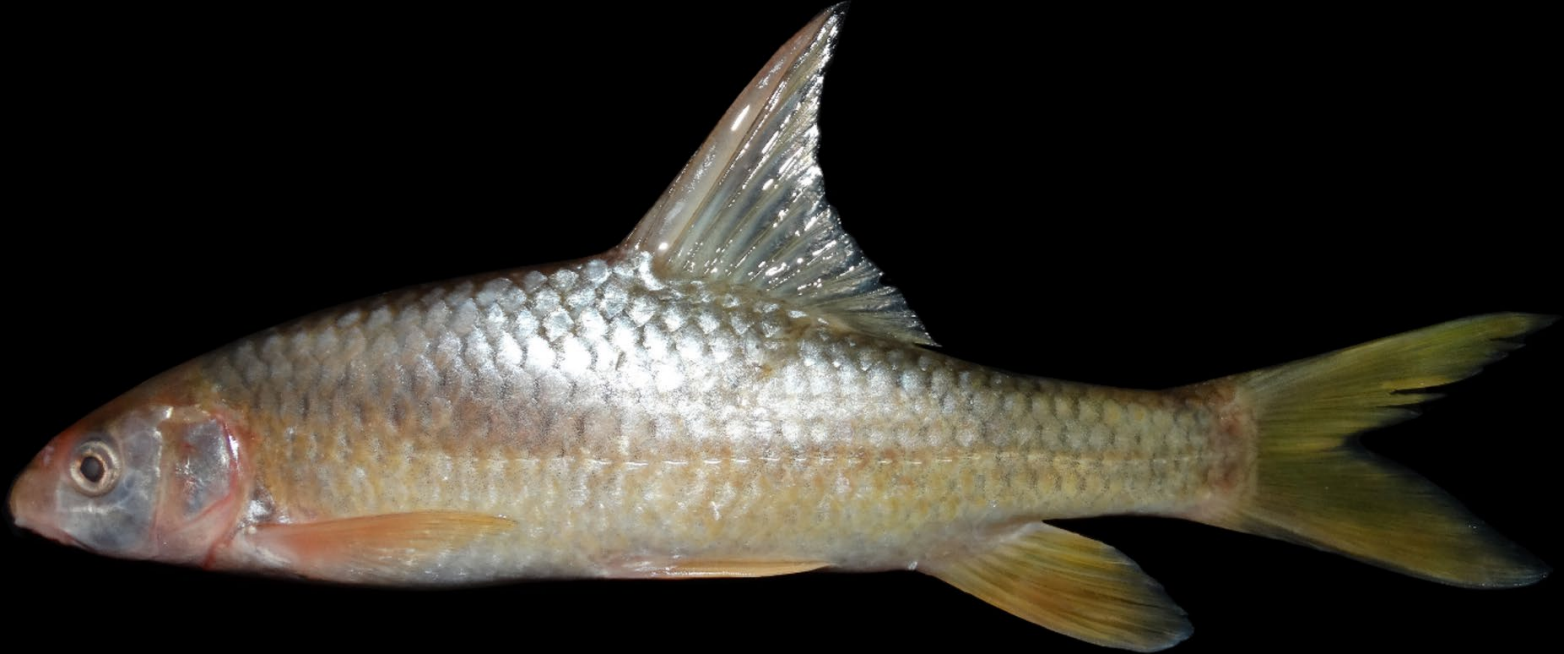
*Carasobarbus saadatii* sp. n.; BIAUBM 8-H, holotype, 188 mm SL; Iran: Karun River, Persian Gulf basin.

**Fig. 16:**
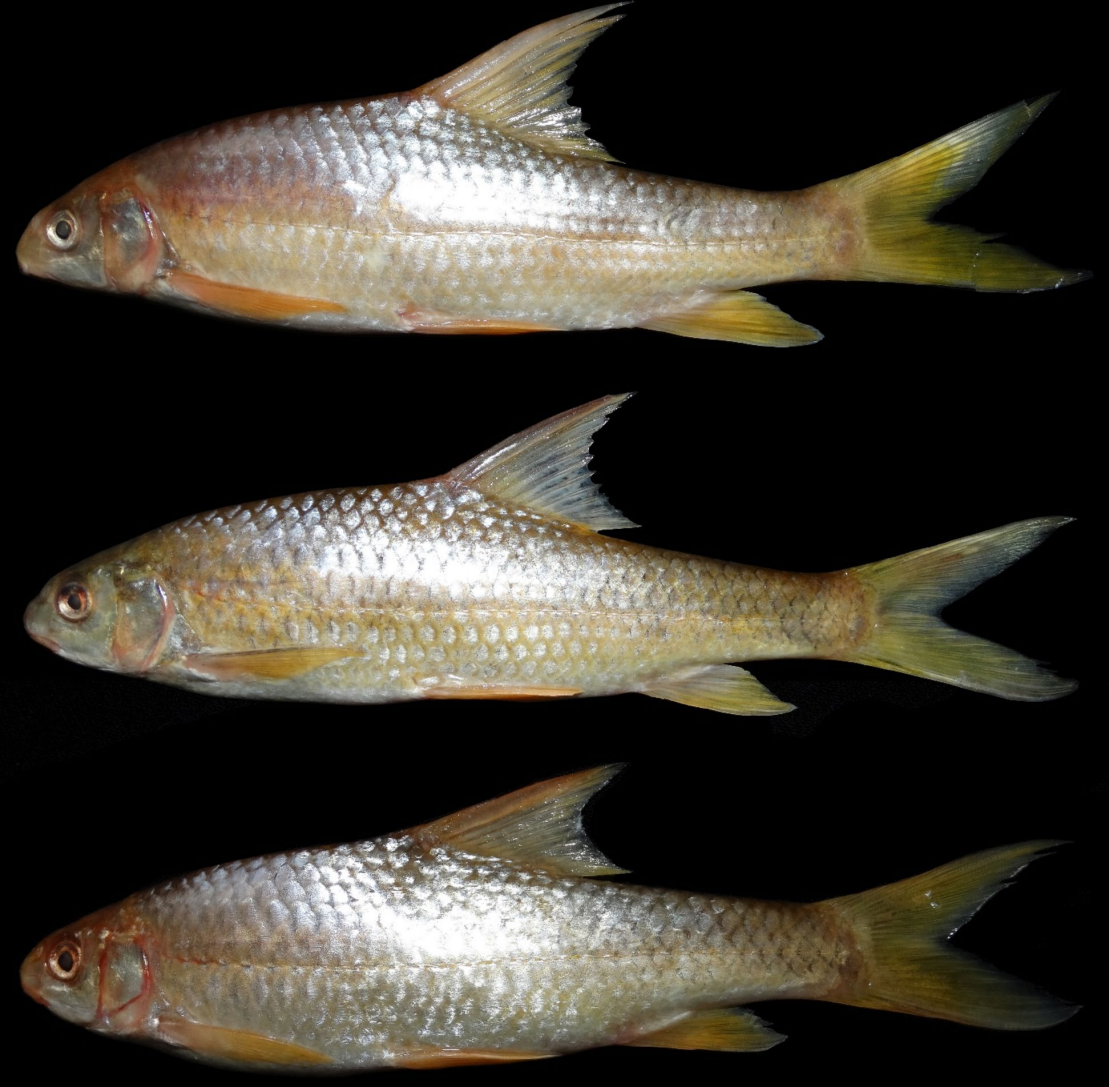
*Carasobarbus saadatii* sp. n., AJRPC 24-P, paratypes, from top: 174 mm SL; 177 mm SL; 188 mm SL; 123 mm SL; Karun River, Persian Gulf basin.

**Fig. 17:**
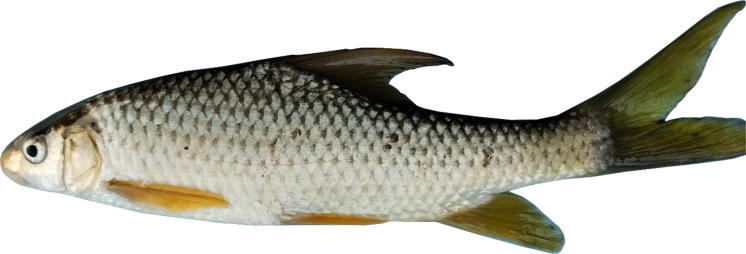
*Carasobarbus saadatii* sp. n.; uncatalogued, 175 mm SL; Iran: Karun River, Persian Gulf basin.

***Carasobarbus saadatii*** **, new species**

(Figs. 15, 16 and 17).

**Holotype**. BIAUBM 8-H, 187.6 mm SL; Iran: Khuzestan prov., Karun River at Gotvand, Persian Gulf Basin, 32.27319, 48.83521.

**Paratypes.** AJRPC 24-P, 4, 122.9–179.4 mm SL; data same as holotype.

**New material used in molecular genetic analysis.** AJRPC-DNA 1860 (COI: PP515189, Cyt *b*: PP548217), 1861 (COI: PP515190, Cyt *b*: PP548218), 1862 (COI: PP515191, Cyt *b*: PP548219), 1863 (COI: PP515192, Cyt *b*: PP548220) same data as holotype.

**Fig. 18:**
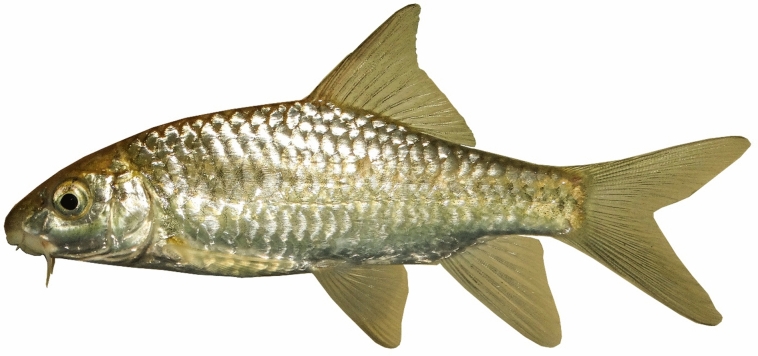
*Carasobarbus sublimus*; VPFC Fahlian 1400.10., 132 mm SL; Iran: Fahlian River.

**Fig. 19:**
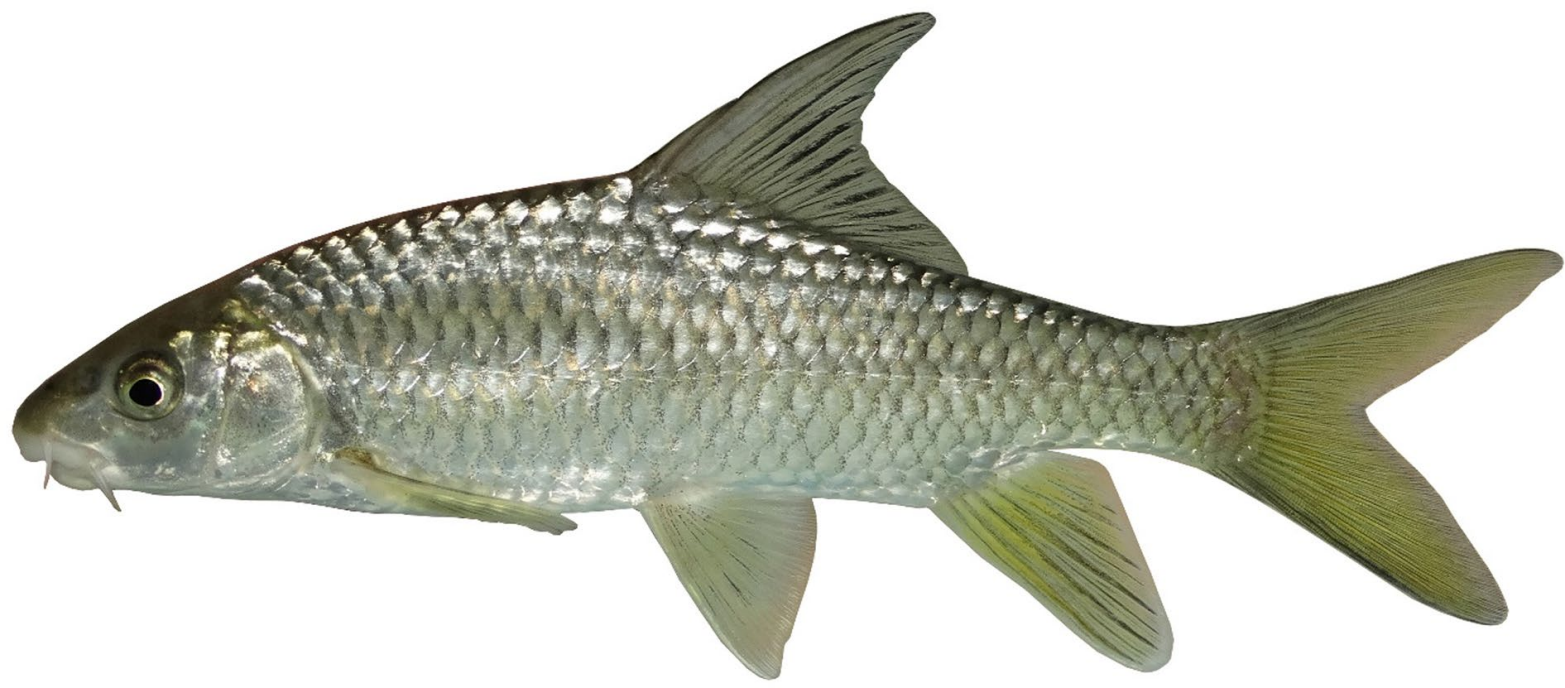
*Carasobarbus kosswigi*; VPFC NeypahnSeyfolah 1400.7., 85 mm SL; Iran: Alvand River, Tigris drainage.

**Fig. 20:**
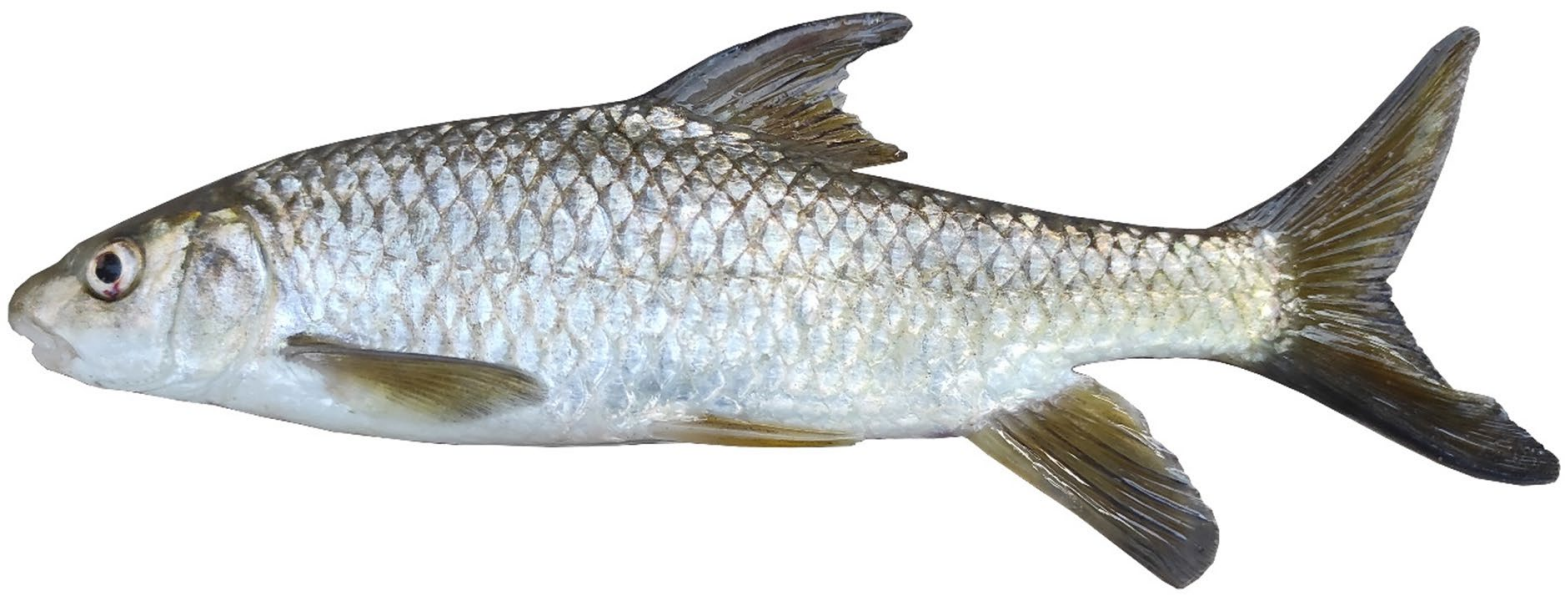
*Carasobarbus kosswigi*; uncatalogued, about 175 mm SL; Türkiye: Tigris River.

**Fig. 21:**
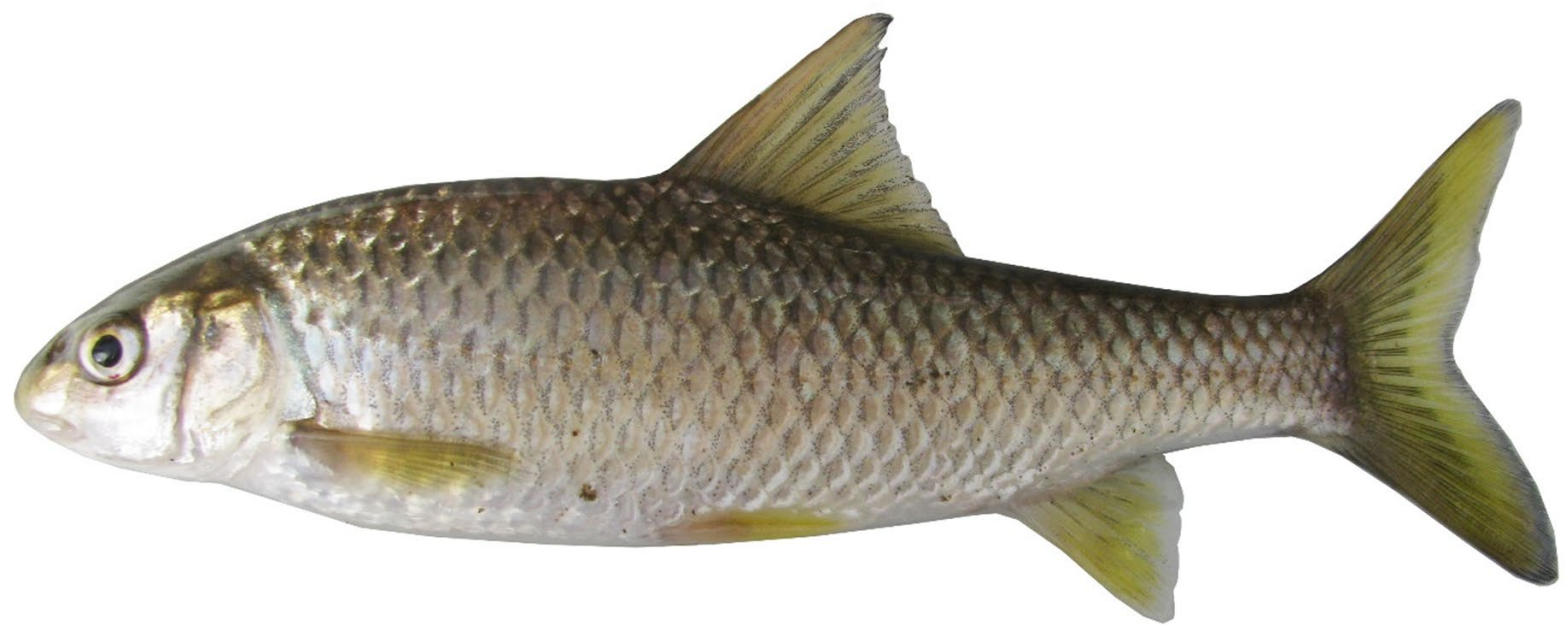
*Carasobarbus kosswigi*; VPFC Hajij 1394.4., 114 mm SL; Iran: Sivan River.

**Diagnosis.** *Carasobarbus saadatii* sp. n. is distinguished from *C. sublimus* (Fig. 18), *C. hajhosseini* sp. n. and *C. kosswigi* (Figs. 19, 20 and 21) by having more scales on lateral line (38–40 vs. 27–37).

**Fig. 22:**
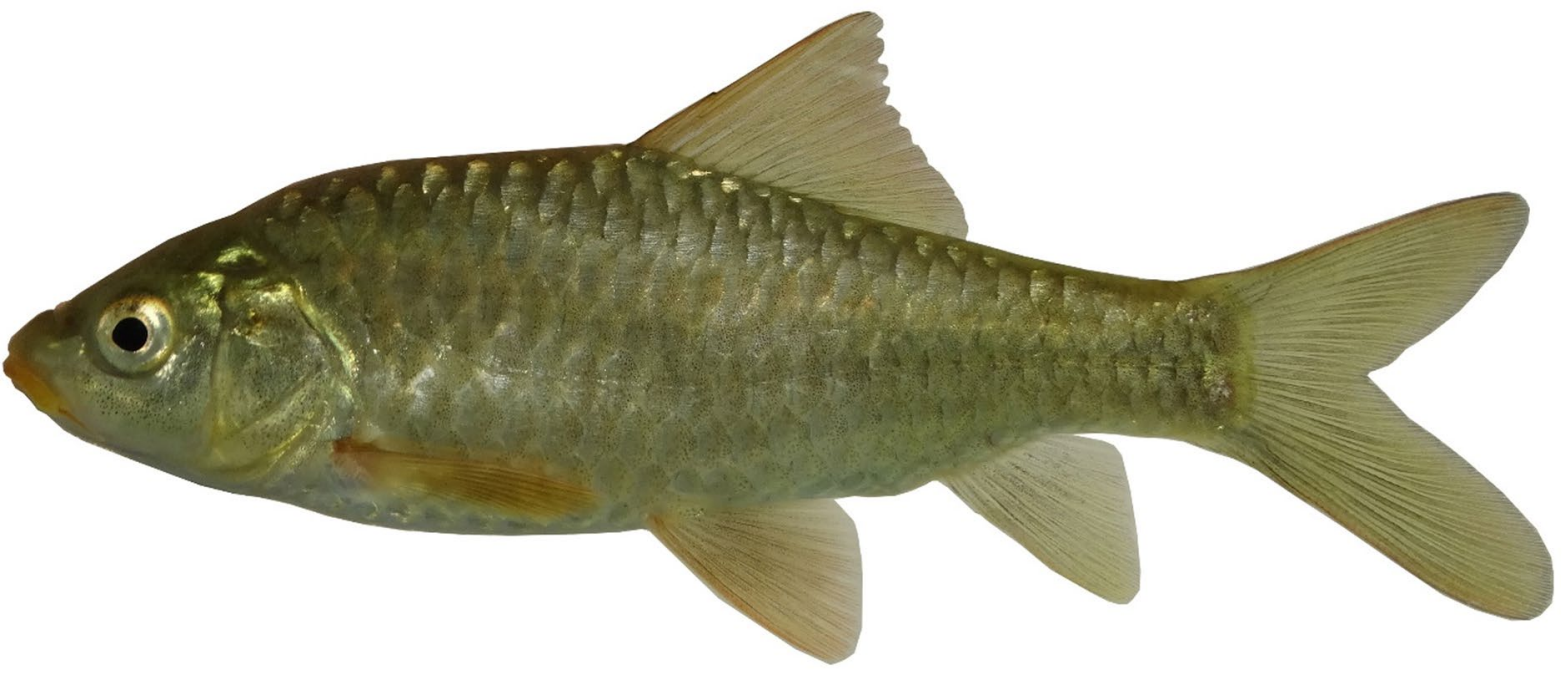
*Carasobarbus luteus*; VPFC SiyahGav 1400.9., 81 mm SL; Iran: Siyah Gav Lake.

**Fig. 23:**
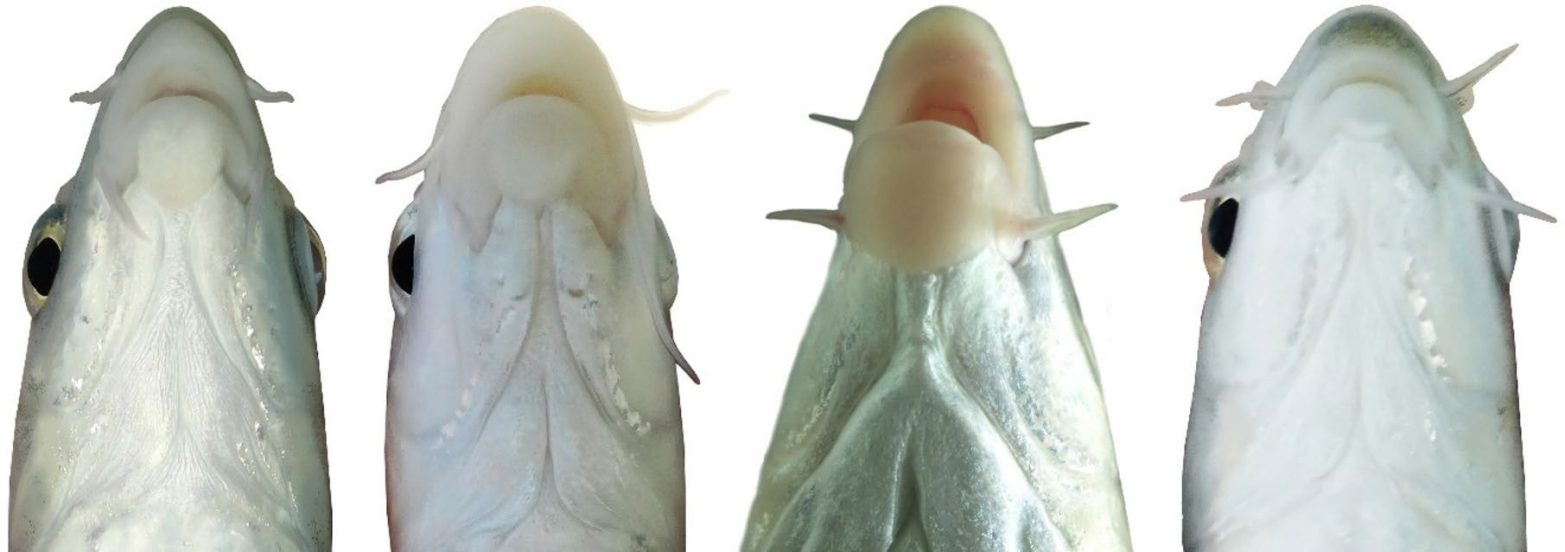
The ventral view of the head. From left to right: *Carasobarbus kosswigi*, VPFC NeypahnSeyfolah 1400.7., 85 mm SL; *C. sublimus*, VPFC Fahlian 1400.10., 132 mm SL; *C. doadrioi* sp. n., uncatalogued, about 150 mm SL; *C. hajhosseini* sp. n., AJRPC 21-P, 137 mm SL.

The new species can be distinguished from *C*. *luteus* (Fig. 22) by having two pair of barbels (vs. one pair), well-developed median lobe on the lower lip (vs. without median lobe) (Fig. 23) and more scales on the lateral line (38–40 vs. 25–30).

### Description

See Figs. 15, 16 and 17 for general appearance, Table 7 for morphometric data. Body moderately high, laterally compressed, without nuchal hump. The greatest body depth at point of origin of dorsal fin. Ventral head profile straight, dorsal profile has a slight to pronounced hump near the nostrils. A rounded keel on back in front of dorsal fin. Head short and narrow. Maximum body depth larger than head length. Triangular axillary scale at pelvic-fin base. Pelvic-fin origin below vertical of last unbranched dorsal fin ray. Caudal fin forked. Tip of anal fin, when pressed to body, reaching to hypural complex. Pectoral fin reaching approximately 70–80% distance from pectoral-fin origin to pelvic-fin origin. Pelvic fin not reaching anus. Eye large, but smaller than snout. Mouth inferior, lips thick and fleshy with a well-developed median lob. Two pairs of barbels, rostral reaches to eye and maxillary reaching to the posterior part of eye.

**Table 7:** Morphometric data of *C. saadatii* sp. n. (holotype BIAUBM 8-H and paratypes AJRPC 24-P; n = 5) and *C. sublimus* (VPFC Zard 1400.9., VPFC Fahlian 1400.10; n = 8) and *C. kosswigi* (FFR 416, FFR 417, FFR 421; n = 17).

Characters	*C. saadatii*					*C. sublimus*				*C. kosswigi*			
	Holotype and paratypes												
	H	Min	Max	Mean	SD	Min	Max	Mean	SD	Min	Max	Mean	SD
Standard length (SL)	188	123	179			72				85	176		
In percent of standard length													
Head length	20.1	18.7	20.1	19.1	0.6	23.1	26.8	25.5	1.7	24.5	27.4	25.7	0.8
Maximum body depth at dorsal fin origin	27.2	27.0	29.9	28.1	1.2	27.1	32.2	29.0	2.5	25.4	29.9	27.8	1.3
Body depth at anal fin origin	18.2	18.0	20.3	19.3	1.0	19.1	22.4	20.8	1.5	17.0	19.9	18.4	1.0
Pre-dorsal length	54.1	48.9	54.1	51.9	2.2	51.7	56.6	54.3	2.1	47.0	53.1	50.5	1.7
Pre-pelvic length	53.1	47.6	53.1	49.6	2.2	51.5	55.5	53.6	1.9	49.2	52.6	51.1	0.9
Pre-anal length	75.1	74.2	76.3	75.1	0.8	74.5	77.8	75.8	1.6	73.9	78.9	75.9	1.4
Dis. betw. pectoral and anal fins	55.1	55.0	57.6	56.4	1.2	46.3	51.4	49.0	2.1	50.2	56.4	53.4	1.4
Dis. betw. pectoral and pelvic fins	29.8	29.5	30.6	29.9	0.4	25.0	28.5	27.2	1.5	25.0	29.7	27.6	1.1
Dis. betw. pelvic and anal fins	25.6	25.6	28.1	26.9	1.1	21.1	23.8	22.6	1.2	18.6	30.1	26.0	2.4
Dorsal fin height	28.2	26.2	29.7	27.9	1.4	21.1	26.1	23.2	2.2	25.3	30.5	27.5	1.2
Anal fin height	22.0	20.4	22.9	21.3	1.1	20.4	27.2	23.5	3.0	17.5	27.4	22.2	3.3
Pectoral fin length	22.1	21.7	24.0	22.6	1.0	20.6	22.3	21.4	0.7	20.2	22.4	21.0	0.5
Pelvic fin length	19.1	18.8	21.3	19.8	1.0	19.1	21.0	20.1	0.8	18.6	20.1	19.3	0.5
Upper caudal fin lobe	29.7	29.7	33.7	31.6	1.7	29.5	34.9	32.8	2.5	27.8	32.0	29.9	1.2
Length of middle caudal fin	9.7	9.7	13.3	11.2	1.4	13.0	16.6	15.2	1.6	11.6	14.1	12.9	0.8
Caudal peduncle length	16.3	15.3	17.1	16.3	0.7	13.3	14.9	14.0	0.7	15.2	17.6	16.5	0.7
Caudal peduncle depth	10.2	10.2	12.1	11.0	0.7	10.9	13.1	11.8	1.1	9.3	10.9	10.2	0.4
In percent of head length													
Snout length	28	26	28	26.6	0.8	29	34	30.6	2.2	36	44	39.8	2.2
Eye diameter	23	20	24	22.2	1.7	22	30	25.3	3.1	16	26	19.2	2.5
Head depth at pupil	60	56	62	59.9	2.2	55	61	57.6	2.5	53	61	57.0	2.3
Head depth at nape	88	88	95	90.8	3.0	78	81	80.0	1.6	65	76	70.7	2.9
Posterior barbel	16	16	21	19.4	2.4	24	35	29.1	4.9	13	20	16.5	1.5
Anterior barbel	19	17	21	19.1	1.9	12	20	17.0	3.4	18	25	20.8	2.0

Dorsal fin with 4 (n = 5) unbranched rays and 10½ (n = 5) branched rays, outer margin deeply concave. Anal fin with 3 (n = 5) unbranched and 6½ (n = 5) branched rays, outer margin straight. Pectoral fin with 14 (n = 2)–15 (n = 3) rays. Pelvic fin with 8 (5) rays. Lateral line with 38 (n = 1), 39 (n = 2), 40 (n = 2) scales. Scale rows between dorsal-fin origin and lateral line 6 (n = 4)–7 (n = 1). Scale rows between anal-fin origin and lateral line 5 (n = 6).

### Coloration

In fresh: Body silverish or cream-white. The back darker than the belly. Upper lateral line scales outlined by dark pigmentation, evident in anterior and fade in posterior. Fins with scattered dark melanophores on rays and membranes. In formalin: Body cream-brown, back darker than belly. No dark pigmentation on anterior and posterior section of scales.

### Distribution

The new species distributed in the lower Karun drainage as well as the Great Zab in the Tigris drainage.

### Etymology

The species is named in honour of Mohamadali Saadati (Mashhad), acknowledging his significant contributions to the taxonomy of freshwater fishes in Iran. He holds the distinction of being the first Iranian Ichthyologist, conducting a systematic study on the taxonomy and distribution of freshwater fishes in Iran in 1977. To this day, his findings continue to be utilized by several Ichthyologists in Iran.

### Habitat

**Fig. 24:**
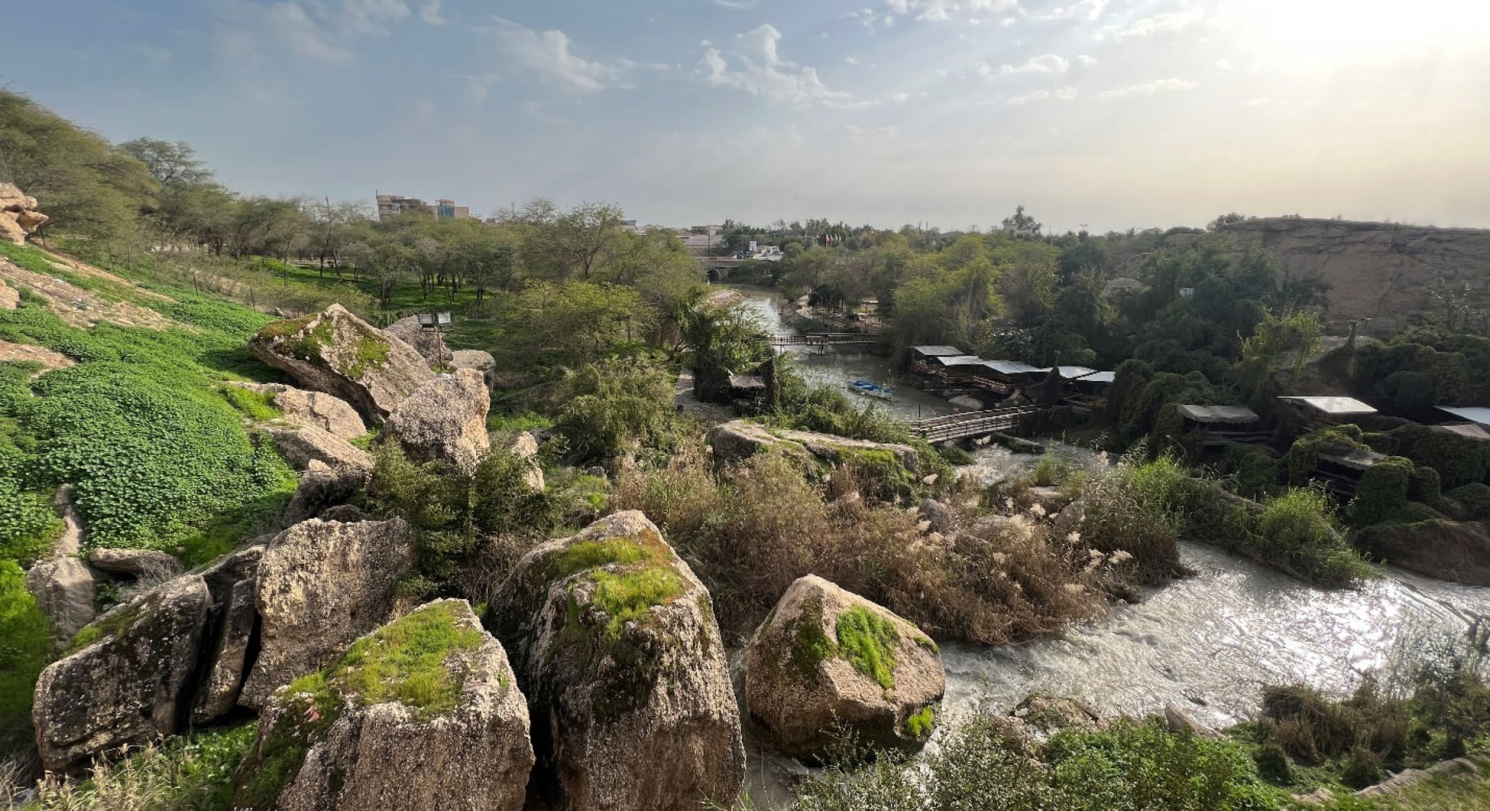
Karun River, between Gotvand and Shushtar, Persian Gulf basin, type locality of *Carasobarbus saadatii* sp. n.

The new species is usually found in the deeper parts of rivers and dam reservoirs, where water flows are slower and there is ample vegetation and cover (Fig. 24). During the summer months, it disperses into faster-flowing waters as well, likely due to warming water temperatures in their typical habitat. It prefers areas along the banks and around islands where tree roots and aquatic plants are accessible. This allows it to forage while remaining hidden among the vegetation to avoid predators. The species appears to be most abundant in the middle and lower Karun. *Luciobarbus barbulus* (Heckel, 1847), *Capoeta aculeate* (Valenciennes, 1844), *Garra rufa*, *Chondrostoma regium*, *Alburnus sellal*, *Squalius lepidus*, *Squalius berak* and *Glyptothorax cous*, were found coexisting with the new species.

## Discussion

In general, fishes of the genus *Carasobarbus* are bottom feeders, with morphological characters specialised for such behaviour. This is especially visible in the differences in the development of their mouth structure and lips. Similar developments have been observed in other species of barbs^26,27^. The lips development in *Carasobarbus* fishes, seems to be a suitable character to separate species^28^. In the newly described species, the *C. hajhosseini* species present the smaller lips (less developed). On the other hand, *C. doadrioi* species, appear to show the most developed lips among them. Check the ventral head view figure (Fig. 23) to compare these differences and observe that both latter mentioned species show both ends of the spectrum. *Carasobarbus saadatii* species also present intermediate lips development similar to *C. sublimus* for example, but we do not have an acceptable picture to show in this work.

Borkenhagen and Krupp^2^ questioned the locality data of the *C. sublimus* specimen (CMNFI 1979-0277), as the morphometric and meristic characters (scales in the lateral line, above the lateral line, and around the least circumference of the caudal peduncle; length of the dorsal, pectoral, ventral, and anal fins) of this specimen are within the range of *C. sublimus* and outside the range of *C. kosswigi*. This discrepancy is unsurprising because the Karkheh population belongs to *C. hajhosseini*, and the range of these characters matches the locality mentioned for this voucher specimen. However, they considered *C. hajhosseini* populations as *C. sublimus*, and *C. doadrioi* and *C. saadati* as *C. kosswigi*, which caused the range of morphometric characters to expand and positioned *C. kosswigi* and *C. sublimus* as paraphyletic in the phylogenetic trees.

In general, nearly all the internal nodes are well resolved in all three datasets (COI, Cyt *b* and concatenated datasets) used in molecular phylogenetic analyses. But as expected, the concatenated dataset resulted in the best resolved tree. Both genetic markers used in the concatenated dataset are mitochondrial markers, i.e. they sare the same evolutionary history. This point out that the improvement in the phylogenetic resolution is most probably due to the increment in the phylogenetic signal coded in a longer sequence fragment. This point underlines the importance of including multiple markers to be able to resolve remaining obscure relationships within the genus. On the other hand, being hexaploid, complicates the inclusion of any nuclear marker in any genetic study in near future^29^. This point is important as some species of the genus (for example *C. luteus*) is widespread in a variety of habitats and therefore will not be surprising to find that different populations does not share the same evolutionary history. This will not be visible without analysing both mitochondrial and nuclear genomic markers.

In the obtained mitochondrial phylogenetic results in the actual study, the only unresolved relationship, is the one between *C. doadrioi*, *C. hajhosseini* and *C. saadatii*. The very short internal branch at this level, when present, shows a potential rapid speciation event, resulting in small number of conserved changes to resolve this relationship. In our results, based on the partial COI gene, two clearly separate clades are formed with both containing sequences identified as *C. harterti* and *C. fritschii*. This is most probably the result of misidentification, or also it can be due to introgression events. As we do not have access ourselves to the material used in this case (genetic material was retrieved from GenBank), we cannot further develop on this and corroborate the identity of each of the clades. On the other hand, using other individuals identified as these two species, they do separate well in the results of the cyt *b* gene dataset, with no further issues. Another possible issue which will need further investigation is the inclusion of samples identified as *C. apoensis* within the *C. luteus* clade, with practically no genetic difference with them. This point was also mentioned in Borkenhagen^28^. Based on this observation we recommend a systematic revision of both *C. apoensis* and *C. luteus* in further studies.

### Comparative materials examined

***Carasobarbus kosswigi*.** Iran: – VPFC NeypahnSeyfolah 1400.7., 1, 85 mm SL; Iran: Kermanshah prov.: Alvand River at Neypahn Seyfolah, Karkheh, 34.408611, 45.586944. – VPFC Hajij 1394.4., 1, 114 mm SL; Iran: Kermanshah prov.: Sirvan River at Hajij, Tigris drainage, 35.15678, 46.32132 (now under dam).

Türkiye: – FFR 416, 17, 124–176 mm SL; FFR 417, 1, 170 mm SL; FFR 421, 4, 129–168 mm SL; Siirt prov.: Botan River at 8 km southwest of Siirt, Tigris drainage, 37.85268, 41.88749.

***Carasobarbus sublimus*.** Iran: – VPFC Zard 1400.9., 2, 72–132 mm SL; Iran: Fars prov., Zard River at Zard Mashin, Marun drainage, 31.37633, 49.72072. – VPFC Fahlian 1400.10., 2, 111–92 mm SL; Iran: Fars prov., Fahlian River at Fahlian bridge, Zohre drainage, 30.18520, 51.52443.

***Carasobarbus luteus*.** Iran: – VPFC Siyahgav 1400.9., 7, 65–95 mm SL; Iran: Ilam prov., Siyah Gav Lake, near Abdanan, Tigris drainage, 32.86564, 47.70155. – VPFC Golabi 1400.10., 1, 130 mm SL; Iran: Fars prov., Golabi spring, near Darab, Kol drainage, 28.78766, 54.37183.

### New material used in molecular genetic analysis

***Carasobarbus kosswigi*.** Türkiye: AJRPC-DNA 1882 (COI: PP515193, Cyt *b*: PP548221), 1883 (COI: PP515194, Cyt *b*: PP548222), Şırnak prov.: Tigris River at 4 km north of Cizre, 37.375610 42.147106; 1884 (COI: PP515195, Cyt *b*: PP548223), Şırnak prov.: Tigris River at Damlarca, 37.404131 42.070865. Iran: AJRPC-DNA 45 (COI: PP515174, Cyt *b*: PP548208), Kermanshah prov.: Alvand River at Neypahn Seyfolah, Karkheh, 34.408611, 45.586944.

***Carasobarbus sublimus*.** Iran: AJRPC-DNA 400A (COI: PP515179, Cyt *b*: PP548213), 400B (COI: PP515180, Cyt *b*: PP548214), Iran: Fars prov., Zard River at Zard Mashin, Marun drainage, 31.37633, 49.72072.

***Carasobarbus luteus*.** Iran: AJRPC-DNA 554B (COI: PP515181) Kermanshah prov.: Alvand River at Neypahn Seyfolah, Karkheh, 34.408611, 45.586944; 707 (COI: PP515184) Khuzestan prov., Karun River at Gotvand, Persian Gulf Basin, 32.27319, 48.83521; Iraq: AJRPC-DNA 1465 (COI: PP515187), Al Najaf prov.: Euphrates River at Kafal, Persian Gulf Basin, 32.22339, 44.36113; 1376 (COI: PP515185), 1377 (COI: PP515186), Maysan prov.: Tigris River at Amareh, Persian Gulf Basin, 31.85783, 47.13605.

## Data Availability

All the specimens obtained in this study are deposited in local publicly accessible (upon request) zoological collections. The genetic data obtained in this study is deposited in NCBI’s GenBank, the accession numbers for each gene marker is mentioned after each specimen’s voucher code.

## Abbreviations

SL: Standard length
HL: Lateral head length
BIAUBM: Babol Islamic Azad University Biological Museum, Babol, Iran
AJRPC: A. Jouladeh-Roudbar personal fish collection, Tehran, Iran
VPFC: S. Vatandoust Personal Fish collection, Qaem Shahr, Iran
FFR: Faculty of Fisheries, Recep Tayyip Erdogan University, Rize, Turkey
