# Author Correction: New insights into the phylogeny of *Carasobarbus* Karaman, 1971 (Actinopterygii, Cyprinidae) with the description of three new species

**DOI:** 10.1038/s41598-024-75851-x

**Published:** 2024-10-18

**Authors:** Arash Jouladeh-Roudbar, Cüneyt Kaya, Saber Vatandoust, Hamid Reza Ghanavi

**Affiliations:** 1 Museum für Naturkunde, Leibniz Institute for Evolution and Biodiversity Science, 10115 Berlin, Germany; 2 Faculty of Fisheries, Recep Tayyip Erdogan University, Rize, Turkey; 3 Department of Fisheries, Babol Branch, Islamic Azad University, Babol, Iran; 4 Department of Biology, Lund University, Lund, Sweden

Correction to: *Scientific Reports* 10.1038/s41598-024-71463-7, published online 18 September 2024

The original version of this Article did not include evidence of registration in ZooBank within the work itself, as required by Article 8.5.3 of the International Code of Zoological Nomenclature^1^.

This published work and the Nomenclatural Acts it contains have been registered in ZooBank with the LSID urn:lsid:zoobank.org:pub:891DC1CD-C71C-4783-B009-F3141E542A9D, urn:lsid:zoobank.org:act:9296233A-FA76-41E7-9814-7D22FCF37CDC, urn:lsid:zoobank.org:act:B056DC31-E811-4B1C-850B-0DE8767A07F5, and urn:lsid:zoobank.org:act:B4B0C801-0C84-4247-B488-767EC4307CF9.

The original Article has been corrected.

## References

[1] International Commission on Zoological Nomenclature (ICZN). *International Code of Zoological Nomenclature* 4th edn, i–xxix, 1–306 (The International Trust for Zoological Nomenclature, London, 1999). 10.3897/zookeys.931.51583 PMC7205856 32405237

